# FHSA-SED: Two-Locus Model Detection for Genome-Wide Association Study with Harmony Search Algorithm

**DOI:** 10.1371/journal.pone.0150669

**Published:** 2016-03-25

**Authors:** Shouheng Tuo, Junying Zhang, Xiguo Yuan, Yuanyuan Zhang, Zhaowen Liu

**Affiliations:** 1 School of Computer Science and Technology, Xidian University, Xi'an, 710071, P.R. China; 2 School of Mathematics and Computer Science, Shaanxi University of Technology, Hanzhong, 723000, P.R. China; Huazhong University of Science and Technology, CHINA

## Abstract

**Motivation:**

Two-locus model is a typical significant disease model to be identified in genome-wide association study (GWAS). Due to intensive computational burden and diversity of disease models, existing methods have drawbacks on low detection power, high computation cost, and preference for some types of disease models.

**Method:**

In this study, two scoring functions (Bayesian network based K2-score and Gini-score) are used for characterizing two SNP locus as a candidate model, the two criteria are adopted simultaneously for improving identification power and tackling the preference problem to disease models. Harmony search algorithm (HSA) is improved for quickly finding the most likely candidate models among all two-locus models, in which a local search algorithm with two-dimensional tabu table is presented to avoid repeatedly evaluating some disease models that have strong marginal effect. Finally G-test statistic is used to further test the candidate models.

**Results:**

We investigate our method named FHSA-SED on 82 simulated datasets and a real AMD dataset, and compare it with two typical methods (MACOED and CSE) which have been developed recently based on swarm intelligent search algorithm. The results of simulation experiments indicate that our method outperforms the two compared algorithms in terms of detection power, computation time, evaluation times, sensitivity (TPR), specificity (SPC), positive predictive value (PPV) and accuracy (ACC). Our method has identified two SNPs (*rs3775652* and *rs10511467*) that may be also associated with disease in AMD dataset.

## Introduction

With the advent of high-throughput sequencing technology, it is possible to measure all of single-nucleotide polymorphisms (SNPs) from thousands of individuals [1]. The genome wide association studies (GWAS), that aim to detect the casual relationship between SNPs and disease status and explore multiple SNPs synergistic effect on diseases status in a population, play a very important role in identifying the causes of disease [2] [3] [4], which have successfully identified many SNP genetic markers associated with a wide range of diseases and quantitative traits [5] [6]. Around 30 schizophrenia associated loci have been identified through GWAS techniques [7–10]. However, it is also an enormous challenge in calculation capability to detect the casual relationship between multi-SNPs and disease status at a whole-genome scale due to the enormous computational burden imposed by a very high-dimensional search space: a brute force search method is infeasible to evaluate the entire multi-locus model in genome wide scale. For identifying multi-locus disease models, there have been a number of algorithms proposed to search the multi-locus models in recent years. These algorithms can be categorized as exhaustive combinatorial search, stochastic search, heuristic search and machine learning based technique [11–12].

The exhaustive search approach, in which all possible multi-locus SNP combinations are evaluated on the strength of their associations with disease states, is very simple and can be realized through a parallel mechanism for detecting SNPs combinations for non-genome-wide association study [13–16]. However, current calculation technique is usually limited, and it is infeasible to detect the multi-locus epistasis models using the exhaustive search algorithm for GWAS.

Heuristic search algorithm [17–20] is an approximate search algorithm, which can expedite the search process by reducing the search space. Stochastic search algorithm [21–22] works by using probabilistic methods to search the optimal solution. However, both heuristic search and stochastic search cannot ensure discovering the global optimal solution. Machine learning based technique [23–25] is also adopted widely in computational biology, which can be categorized as classification for difference analysis and regression analysis, which is usually combined with feature selection technique for selecting a group of features (such as SNPs, genes) that affect significantly the phenotypes or traits, but it can not determine the true causal relationship between genotype and phenotype.

In recent years, swarm intelligent optimization algorithms, inspired by natural phenomenon or biological system, have attracted considerable attention for genetic interactions [1, 4, 26–29]. For example, AntEpiSeeker [29] introduced a two-stage ant colony optimization (ACO) algorithm for the detection of epistatic model. M Aflakparast *et al*. (2014) [30] proposed a cuckoo search epistasis (CSE) algorithm which combined Bayesian scoring with cuckoo search (CS) algorithm [31] for detecting the multi-locus disease-causing models. Jing and Shen (2014) [4] proposed a Multi-objective Ant Colony Optimization algorithm for SNP Epistasis Detection (MACOED), in which both Bayesian network scoring and logistical regression scoring are combined as evaluation criterions for SNP interactivities. However, these methods have drawbacks on low detection power and high computation cost.

It is very important to develop or choose appropriate methods for identifying the multi-locus disease-causing models for genome-wide study. There has been remarkable activity in the development of methodology (e.g. Bayesian methods, regression-based methods, linkage disequilibrium (LD) and haplotype-based methods) [32] for the detection of epistasis in the past ten years. However, they perform inconsistently usually with different disease models [4] because they were conceived merely based on part of detective models of epistasis. Some multi-objective detection methods were proposed to improve the performance for detecting the multi-locus epistasis models, such as multi-filter enhanced genetic ensemble (MF-GE) system [23] and multi-objective ant colony optimization algorithm (MACOED). the MF-GE algorithm requires diverse and accurate classifiers to achieve better accuracy and requires configuring parameters properly for each classifier, which is a very large challenge for MF-GE method; MACOED simultaneously employs the Bayesian-based K2-score and regression-based AIC-score as evaluation indexes in the filter stage, in which, however, the two-fold scoring method would increase the computation burden and make some models failed to pass the screening stage due to overly strict evaluation methods. Although the two-fold scoring method could decrease the false positive rate (Type I error) in MACOED, it is apparent that the false negative rate (Type II error) increases; in addition, the regression-based AIC-score methods require an iteration process to optimize the regression coefficients, which is often computationally unaffordable for SNP datasets with very large number of markers. To tackle these drawbacks (preference to some types of disease models, high computation cost), we propose a two stages (screening and testing) intelligent search algorithm named FHSA-SED (Harmony Search Algorithm with two scoring functions for SNP Epistasis Detection)” to detect two-locus disease models. To quickly identify various disease models, in the FHSA-SED algorithm, two evaluation criteria (Bayesian network based K2-score and Gini-score) are employed to enhance the ability for identifying various disease models, Harmony Search Algorithm (HSA) is improved to speed up the process of detecting disease models and a local search algorithm with two-dimensional tabu table is presented to avoid repeatedly evaluating (overcoming the premature convergence) some disease models which have strong main effects.

In this study, our central goal is to detect as various disease models as possible, and to enhance the power of identifying disease models by employing two complementary methods (K2-score and Gini-score). Our method is divided into two stages: in the 1^st^ stage, we want to quickly obtain some most likely two-locus disease models (candidate solutions) using harmony search algorithm; in the 2^nd^ stage, we adopt the G-test method to test the candidate solutions.

Some terms (Joint effect, Evaluation times, Computation time and two-locus disease model) are explained in Box 1.

**Box 1**

**Terms:**

1. **Joint effect (Synergy effect)** denotes *k* SNP locus act jointly to have a particular phenotypic effect, which includes additive effect, statistical interaction effect and so on.
2. **Evaluation times** represent the number that *k-locus* models are evaluated using Bayesian scoring criterion and Gini scoring criterion.
3. **Computation time** denotes the time spent executing algorithm in the program.
4. **two-locus disease model** is defined as by penetrance table, in which a two-way SNP genetic combination is referred to as collective association with the dichotomous phenotype (disease status) if the genotype distribution at the two SNPs is different significantly between cases and controls, and it may be responsible for significantly increasing the risks of complex diseases [33–34].

### Outline

**Fig 1:**
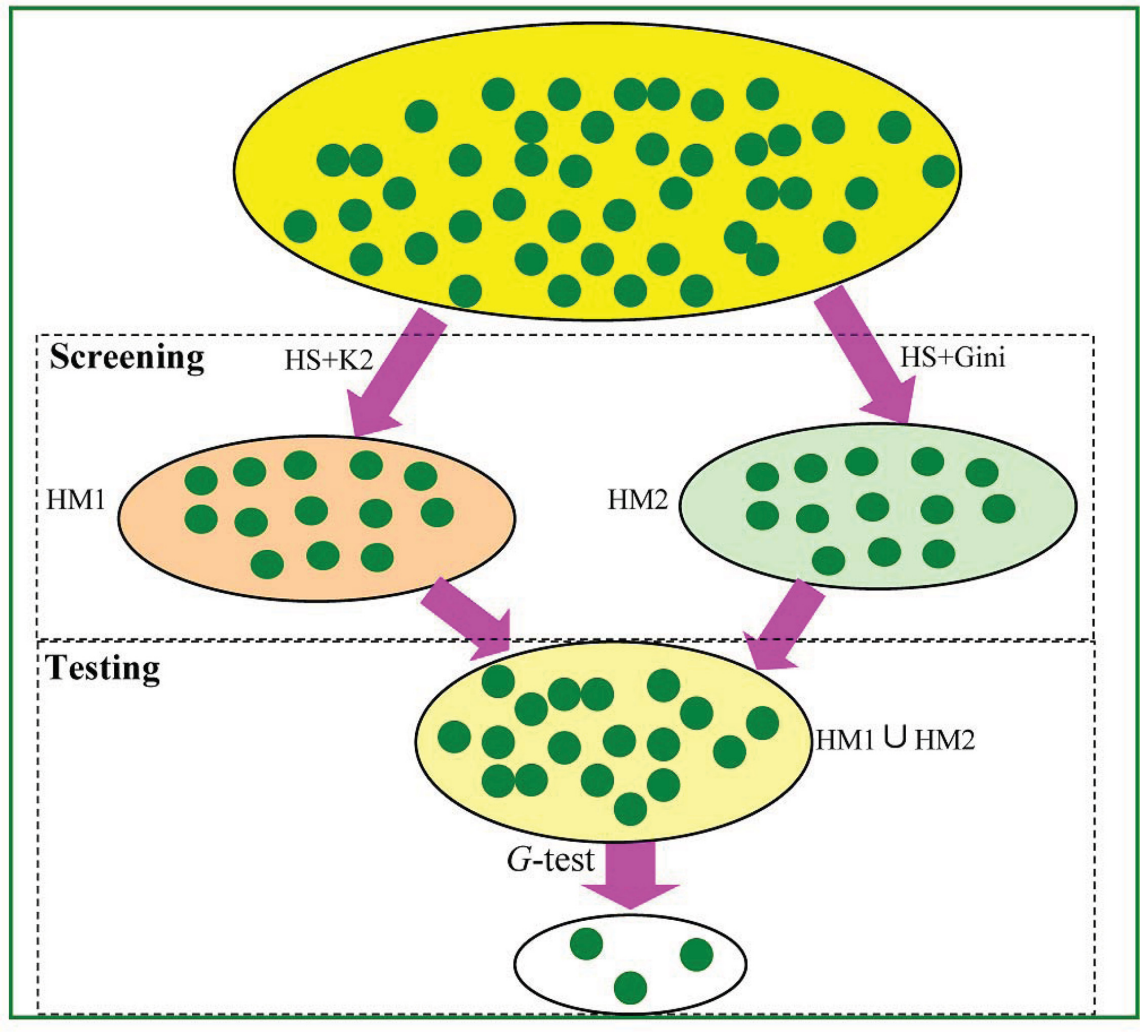
The flow chart of FHSA-SED algorithm. ① Yellow ellipse consists of the entire two-SNP combinations that have not been filtered. ② Orange ellipse contains the two-locus models with highest K2-score, which are the filtered results in 1^st^ stage. ③ Light green ellipse contains the two-locus models with highest Gini-score, which are the filtered results in 1^st^ stage. ④ Pink ellipse is the union of HM1 and HM2. ⑤ Final output results which have passed the G-test are in white ellipse.

A flow chart of our method is illustrated in **Fig 1**, in which the detection process of two-locus disease models in FHSA-SED algorithm is divided into two stages: “screening” and “testing”. In the screening stage, an improved harmony search algorithm (HSA) (Z.W. Geem, 2001) [35] is employed to search two-locus models that might be associated with phenotype, and two criteria (Bayesian network based K2-score and Gini-score) are respectively used to evaluate the causality between the two-locus models and phenotype. Some two-locus models with highest K2-score are stored in harmony memory HM1, and some models with highest Gini-score are stored in HM2. Next, HM1 and HM2 are merged into a union set HM (= HM1∪HM2) as shortlisted candidates. In the Testing stage, these shortlisted candidates are further checked using a *G*-test statistical method.

## Methods

Let a set of SNP variables *X* = {*x*_1_,*x*_2_,⋯,*x*_*N*_} indicate *N* SNP markers for *L* individuals (samples), *Y* be the phenotype variable with values of {*y*_1_,*y*_2_,⋯,*y*_*J*_}; we represent the homozygous major allele, heterozygous allele and homozygous minor allele as 0, 1 and 2, respectively. For a *k*-loci combination model, *I* denotes the number of genotype combinations (there are 3^*k*^ SNP genotype combinations), *J* is the number of phenotype states *Y* (which is equal to 2 for a case-control dataset). *n*_*i*_ is the number of cases in the dataset with SNP nodes taking the *i-*th genotype combination, *n*_*ij*_ represents the number of cases that belong to the phenotype state *j* where the *k*-way SNPs variables have *i*-th genotype combination.

### Bayesian network scoring criterion and Gini index criterion

It is a vital factor to design new effective method for identifying the disease models successfully. Some existing methods [4] [32] usually prefer one type of disease models to others. To tackle the preference problem to various disease models, we employ two evaluation criteria (Bayesian network based K2-score and Gini-score) to improve the power for identifying various disease models.

#### Bayesian network scoring criterion

A Bayesian network (BN) is a kind of statistical model which represents a set of random variables and their conditional dependencies by using a directed acyclic graph (DAG). In the DAG, nodes denote random variables, and edges represent conditional dependences between two linked nodes.

There are more than 20 kinds of BN models [22] [36–37] that have been developed to find causal relationships, perform explanatory analysis, describe the causal influence and make predictions. In GWAS studies, BN model is also used to detect the interaction effect among SNP markers, which can represent the causal relationship between genetic variants and disease status.

In a DAG of Bayesian network for representing the relationships of SNP markers and disease states, there are only directed edges linking from the SNP markers to diseases status, and there is no edge connected from disease state to SNP markers and also no linkage among SNP markers. In the DAG, if and only if SNP *x*_*i*_ is a direct cause of phenotype state *y*_*j*_, there is a direct edge linking from node *x*_*i*_ to phenotype *y*_*j*_.

According the theorem 1 that is given in [38] *(*more detail interpretation about Bayesian network scoring method are introduced in **S1 File)**, the K2-Score based on Bayesian network scoring criterion [37] can be described as Eq (1),

$$K2-Score=\prod_{i=1}^{I}\left(\frac{(J-1)!}{(n_{i}+J-1)!}\prod_{j=1}^{J}n_{ij}!\right)$$ (1)

#### Gini index criterion

Gini index (Gini coefficient) is a measure of statistical dispersion (http://en.wikipedia.org/wiki/Gini_coefficient#cite_note-1) [39–42], which can be used to measure the impurity of a data partition or the inequality among values of a frequency distribution.

For a binary classification case-control problem, the Gini index is a diversity index [43] which is defined as Eq (2).

$$Gini-score=\sum_{i=1}^{I}P_{i}\cdot \left(1-\sum_{j=1}^{J}p_{i,j}^{2}\right)$$ (2)

where, *p*_*i*,*j*_ (pi,j=nijni) is the estimated probability that the *i*-th genotype combination actually associated with phenotype *y*_*j*_. $\left(1-\sum_{j=1}^{J}p_{i,j}^{2}\right)$ means the estimated probability that genotype combination is misclassified as phenotype *y*_*j*_. *P*_*i*_ (Pi=niL) is the percentage of *i*-th genotype combination in sample set.

(Please see the computational process of an example in **Table A1** in **S1 File**.)

### Proposed FHSA algorithm for two-locus disease model detection

Detecting multi-locus models at a whole-genome scale is a non-trivial task since it takes too much time to detect all models from hundreds of millions of SNPs. In this approach, we propose a fast harmony search algorithm (HSA) to accelerate the detection process of disease models, without an exhaustive search.

Standard HSA (*see* **S2 File**) [35] is a meta-heuristic algorithm, which mimics the process of improvising a musical harmony. Compared with traditional mathematical optimization algorithms, HSA does not require substantial gradient information and is not dependent to initialization, making it widely applied in the fields of combinatorial optimization.

In the standard HSA, harmony memory is a set of harmonies. By evaluating their fitness with some criterion, some harmonies in the set are substituted with some other harmonies which are supposed to be with more fitness. Such a process continues until some finishing criterion is satisfied.

In the proposed FHSA-SED algorithm, each harmony denotes a *k-way* (*k*-locus) model that is a combination of *k* different SNP markers (*k* = 2 in this study) and we employ two harmony memories: HM1 and HM2. The harmony in HM1 is evaluated with Bayesian network scoring criterion, and the Gini scoring criterion is used to evaluate the harmony in HM2. [**Fig B1 in S2 File** presents the flow chart of fast harmony search algorithm (FHSA)]. The pseudo code of FHSA is as **Algorithm-1**.

**Algorithm-1:**harmony search algorithm for SNP Epistasis Detection with two scoring criterion (K2-Score and Gini-Score)

    **Input**: maximum model evaluation times (MMEs) of SNP-pairs model, HS parameters: HMCR, PAR and HMS

    **Output**: HM1, HM2, and fitness values of each harmony in HM1 and HM2

1. **Initialize harmony memory HM1 and HM2 randomly.**

    For I = 1:HMS

        HM1(I, 1:*k*) = (*r*_1_,*r*_2_, …,*r*_*k*_); //*r*_*i*_ ∈ {1,2,⋯,*N*}(*r*_1_ < *r*_2_ < … < *r*_*k*_),

        HM2(I, 1:*k*) = (*s*_1_,*s*_2_, …,*s*_*k*_); //*s*_*i*_ ∈ {1,2,⋯,*N*} (*s*_1_ < *s*_2_ < … < *s*_*k*_)

    End

2. **Calculate the fitness value of each harmony in HM1 using Bayesian network scoring function (*f***_***1***_**), and the fitness value of each harmony in HM2 using Gini scoring function (*f***_***2***_**), respectively.**

    For I = 1:HMS

        Score1(I) = *f*_1_ (HM1(I, 1:*k*)); 

        Score2(I) = *f*_2_ (HM2(I, 1:*k*)); 

    End

3. **Generate a new harmony H**_**new**_ **as follows:**

    for i = 1:*k*

      if rand(0,1)<HMCR

        *a* = ⌈*rand*(0,1)×HMS×2⌉;

        if a<HMS

          H_new_(i) = HM1(a, i);

          if rand(0,1)<PAR

            H_new_(i) = H_new_(i) + (*rand*(0,1)−0.5)×|HM1(idbest1,i)−HM1(r_1_,i)|;

          end

        else

          H_new_(i) = HM2(a-HMS, i);

          if rand(0,1)<PAR

            H_new_(i) = H_new_(i) + (*rand*(0,1)−0.5)×|HM2(idbest2,i)−HM2(r_2_,i)|;

          end

        end

      else

        H_new_(i) = ⌈*rand*(0,1)×*N*⌉;

      end

    end

    If H_new_ has been visited before

        execute the local search algorithm in the neighborhood of H_new_;

    end

4. **Calculate the fitness of H**_**new**_ **using scoring functions *f***_***1***_ ***and f***_***2***_ **respectively:**

    *score*1 = *f*_1_(H_*new*_), *score*2 = *f*_2_(H_*new*_);

**5. Determine whether H**_**new**_ **can replace the worst harmony in HM1 or HM2:**

  if score1 is better than Score1(idworst1)

    HM1(idworst1,:) = H_new_;

  end

  if score2 is better than Score2(idworst2)

    HM2(idworst2,:) = H_new_;

  end

    **6. If termination conditions meet, output HM1 and HM2, otherwise, turn to step 4.**

    *In algorithm1*, *r*_*1*_ *and r*_*2*_ *are random integer between 1 and HMS; idbest1/ idworst1 denotes the index of best/ worst harmony in the HM1; idbest2 and idworst2 denote the indexes of best and worst harmones in the HM2*, *respectively*.

#### Local search algorithm for FHSA

As a heuristic search algorithm, HSA is also easy to trap into a local search and repeatedly evaluate some solution (sampling with repetition) in solving the combinational optimization problems, which causes time-consuming due to these repeated calculation (repeated sampling). To tackle this problem, we establish a tabu table (TT) to store the evaluation state of each SNP-pair (If a SNP-pair has not been evaluated, its evaluation state equals '0', otherwise, it is '1'.) and a local search algorithm is proposed to discover new disease models that have not been evaluated. The TT is different from frequently-used linear tabu list, which is a two-dimensional table for marking the state of each two-locus model, if a two-locus model has been evaluated, its corresponding value on TT is set to "1"; otherwise it is equal to "0". The advantage of the two-dimensional tabu table compared to linear tabu list is that it can get the evaluation state of each two-locus model using one times search (time complexity is O(1)); however, linear tabu list requires a sequential search whose time complexity is O (n).

**Fig 2:**
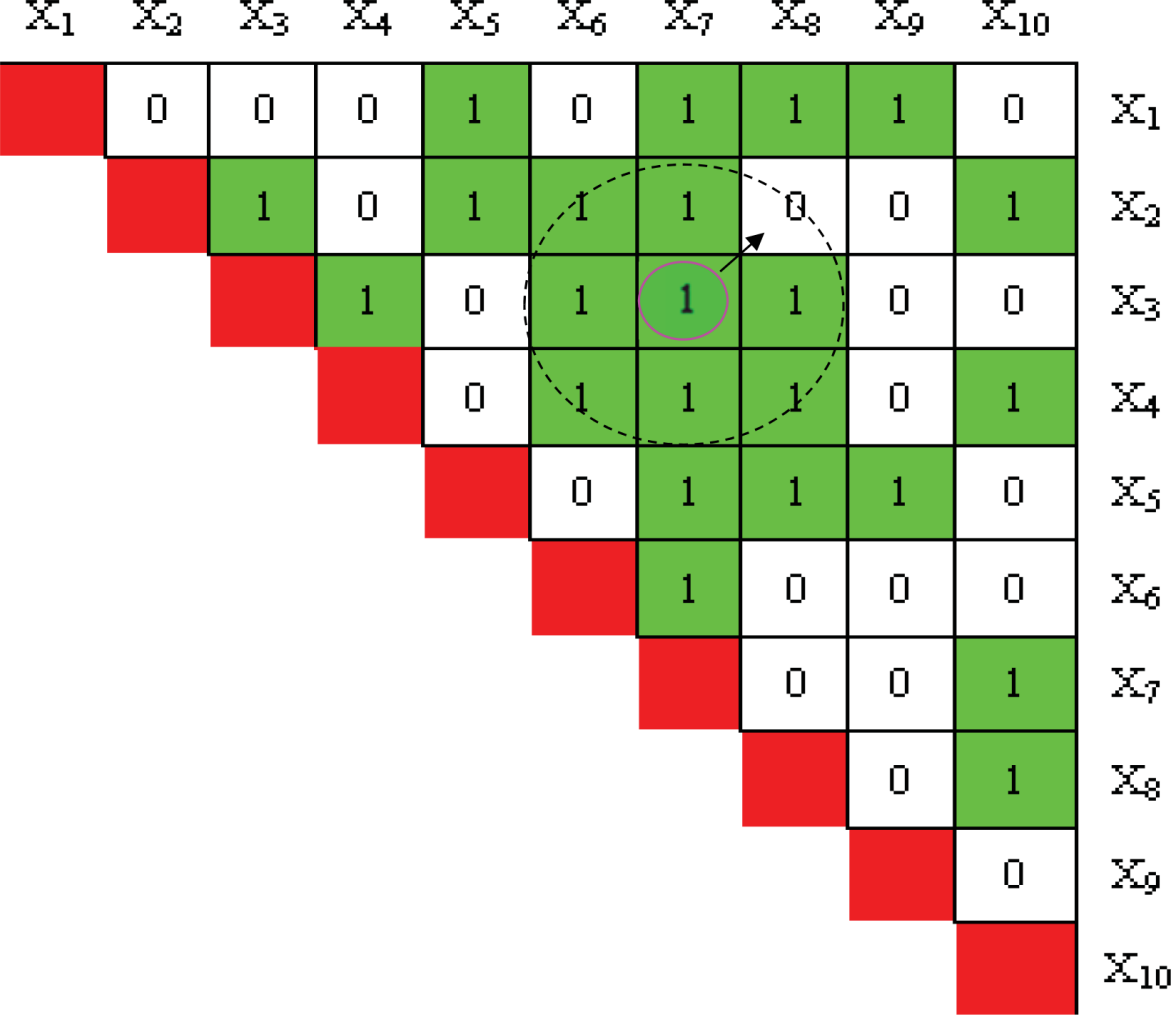
Local search algorithm based on two-dimensional tabu table (TT). In Fig 2, X_i_ is the i^th^ SNP locus, '1' denote the two-locus model has been evaluated. '0' is otherwise.

Local search algorithm is used to obtain a closest solution (that has not been visited) in the neighborhood of current solution, for example **Fig** 2, if a new generated solution H_new_ = (X_7_, X_3_) has been visited, then one of the nearest solutions that have not been evaluated will replace it as new solution H_new_ = (X_8_, X_2_) to be evaluated. The local search algorithm has two advantages: First, it can avoid evaluating the same one two-locus model twice; second, it can achieve the same performance as exhaustive search if we set the maximum model evaluation times (MMEs) equal to $\left(\begin{array}{c} N\\ k \end{array}\right)$. Therefore, the proposed FHSA algorithm is a global search algorithm for detecting two-locus disease model.

**Fig 3:**
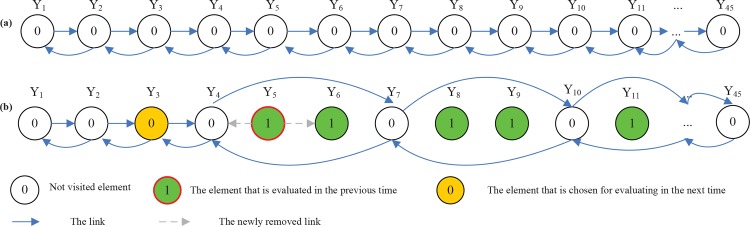
Doubly linked Table as tabu table (TT). All adjacent elements are linked each other. (b) When an element is just evaluated, one of near solutions of the element is selected with a random step for evaluating in the next time.

However, when most of the elements of TT have been marked, the efficiency of local search algorithm will decrease because most of solutions nearby current solution H_new_ have been evaluated, which will increase search times for near solutions. Thus we transform the two-dimensional TT into a link Table. For each element TT_ij_ in two-dimensional Table can be denoted with a link table element Y(k). The transformational formula is expressed as Eq (3). The **Fig** 2 can be transformed as **Fig** 3.

$$Y\left(N\times (i-1)-\frac{i(i-1)}{2}+j-i\right)\leftarrow {\text{TT}}_{ij}$$ (3)

where, N is the number of SNP.

In **Fig** 3, we establish a doubly linked list for storing *Y*, in which adjacent elements in row major order are linked with doubly links at first (see **Fig** 3(A)). When one element (solution) has been evaluated, it will be removed from the doubly linked list. Meantime, in the doubly linked list, one of near elements of the solution will be chosen with a random step *BW*, which is evaluated in the next time (see **Fig** 3(B)). The *BW* is changed dynamically with the increasing of iterations, which is expressed as Eq (4). It can be seen from Eq (4) that the *BW* is between 1 and 10. In the beginning stage, there are most of the elements that have not been evaluated, thus at this time it has a large probability that *BW* possesses a large value. Conversely, in the later stage, it has a large rate that *BW* possesses a small value.

BW=min(10,max(rand(0,1)×#E,1)) (4)

where, #E denotes the number of elements having not been evaluated in doubly linked list.

### G-test

G-test is a likelihood-ratio test that is being progressively applied in different significance tests (http://en.wikipedia.org/wiki/G-test#cite_note-2) [44]. The G-test and chi-squared (*χ*^2^) test will lead to the same conclusions for a reasonable sample size with the Person chi-squared tests. However, the Pearson *χ*^2^ test is inferior to the approximation to the theoretical chi-squared distribution for the G-test [42]. And for testing goodness-of-fit, G-test statistical method is more efficient than Pearson *χ*^2^ test method [45–47].

The general formula for the value of *G* is as follows

$$G=2\sum_{i=1}^{}O_{i}\cdot \ln\frac{O_{i}}{E_{i}}$$ (5)

where *O*_*i*_ is the observed frequency, *E*_*i*_ is the expected frequency under the null hypothesis, ***ln*** denotes the natural logarithmic function.

For *k*-loci model detection, an *I*×*J* contingency table requires be adopted for calculating the *G* value with the follow formula

$$G=2\sum_{i=1}^{I}\sum_{j=1}^{J}O_{ij}\cdot \ln\frac{O_{ij}}{E_{ij}}$$ (6)

where, *O*_*ij*_ and *E*_*ij*_ are respectively the observed numbers and expected number of genotypes when phenotype takes the state *y*_*j*_ and genotypes take *i*-th *k*-combination. We can get the observed number *O*_*ij*_ from dataset by using simple counting statistics method. The expected number *E*_*ij*_ of genotype frequency could be obtained according to Hardy-Weinberg principle [48].

The null hypothesis is that the *k*-combination of SNP set has no association with the phenotype. If the *P*-value of the *G*-test statistic is smaller than a significance level *α*_0_, the alternative hypothesis is accepted, which means the *k*-combination of SNPs has a certain association with phenotype. In order to control false positive rate (Type I error rate), we adopt Bonferroni-corrected significance level $\alpha =\alpha_{0}/\left(\begin{array}{c} N\\ k \end{array}\right)$ to deal with multiple testing. Because sometimes the number of some genotypes equals zero or very small (less than *ε*, *ε* is a small integer) we do a minor modification for calculating G-test value as follows,

$$\begin{array}{l} G=2\sum_{i=1}^{I}\sum_{j=1}^{J}O_{ij}\cdot P_{ij}\\ P_{ij}=\{\begin{array}{c} \ln\frac{O_{ij}}{E_{ij}},O_{ij}>0\\ 0,otherwise \end{array} \end{array}$$ (7)

The degree of freedom *d* (*d* = (*I*−1)(*J*−1)) is also modified as follow:

**For each** *i*(*i* = 1,2,⋯,*I*)

**If** $\sum_{i=1}^{I}O_{ij}<\varepsilon$, **then** the degree of freedom *d* = *d*−1.

End

## Experiments and Results

### Parameters and Environments Setting

To investigate of FHSA-SED algorithm, we evaluated its performance using 82 simulation datasets with different type of disease models and compared its performance with two excellent intelligent optimization algorithms (MACOED, CSE). The MACOED and CSE algorithm have advantages over AntEpiSeeker, BEAM and BOOST on the detection of multi-locus disease models in terms of power, sensitivity (true positive rate: TPR) or specificity (**SPC**) (true negative rate: **TNR**). The Matlab source codes of MACOED[4] and CSE[30] algorithms can be downloaded from http://www.csbio.sjtu.edu.cn/bioinf/MACOED/ and http://lbb.ut.ac.ir/Download/LBBsoft/CSE separately, in which we made some minor revisions on the source codes of two methods in order to perform a fair comparison; the main body of the source codes was unchanged.

In the experiments, parameters setting for the compared algorithms are shown in Table 1. To make a fair comparison, we set the same termination condition and the same runtime environment for three compared algorithms, where maximum model evaluation times (MMEs) is less than the number by using exhaustive search algorithm. All the experiments were performed on Windows XP 64 system with Intel(R) Xeon(R) CPU E5504 @2.0GHz, 8 GB memory, and all the program codes were written in MATLAB R2014b (the source code of FHSA-SED is in **S5 File**).

**Table 1:** The parameters setting of the three algorithms.

Algorithms	Parameters
**FHSA-SED**	HMCR=0.9; PAR=0.35; ||HM1||=100; ||HM2||=100; P-value=0.01/$C_{N}^{k}$; ||∙|| denotes the size of set; MMEs = 4500 for 100SNP markers; MMEs = 300000 for 1000SNP markers
**MACOED**	*τ*_0_ = 1; *T*_0_ = 0.8; *β* = 0.9; *λ* = 2; Ant number =100;P-value=0.01/$C_{N}^{k}$; MMEs = 4500 for 100SNP markers; MMEs = 300000 for 1000SNP markers
**CSE**	*MaxLe’vyStepSize* = 1; Number of SNPs in each Group is 5; Fraction of eggs discarded each generation is equal to 0.25; The number of nest equals 30; MMEs = 4500 for 100SNP markers; MMEs = 300000 for 1000SNP markers

### Performance evaluation criteria

In order to investigate the performance of the FHSA-SED algorithm comprehensively on detecting two-locus disease models which is associated with disease states, we adopt seven metrics: **power**, **evaluation times**, **computation time**, sensitivity (true positive rate: TPR), specificity (**SPC**) (true negative rate: **TNR**), Positive predictive value (**PPV**) and Accuracy (**ACC**).

(1) **Maximum Model evaluation times** (**MMEs):** in the experiment, we set Maximum Model evaluation times (MMEs) of SNP combinations as the terminal condition of algorithm, in other words, the harmony search algorithm will be terminated if the current evaluation times of two-locus models have been larger than MMEs. If the known disease-causing models have been found, the searching algorithm would be terminated early, the number that two-locus models have been evaluated currently is defined as Model evaluation times (**MEs**) and the elapsed time from start to end is denoted as computation time.

(2) The TPR, SPC, PPV and ACC are defined as follows

$$\begin{array}{l} \text{TPR}=\text{TP}/(\text{TP}+\text{FN})\\ \text{SPC}=\text{TN}/(\text{FP}+\text{TN})\\ \text{PPV}=\text{TP}/(\text{TP}+\text{FP})\\ \text{ACC}=(\text{TP}+\text{TN})/(\text{TP}+\text{TN}+\text{FN}+\text{FP}) \end{array}$$ (8)

where TP, FP, TN and FN denote the number of true positives, number of false positives, number of true negatives and number of false negatives, respectively.

The TPR, SPC, PPV and ACC in this study are employed to measure the statistical precision of hypothesis testing method for having found disease-models in the screening stage. The TP is equal to the number of disease-models that have passed threshold of testing method, FN is the number of disease-models failed to pass the threshold of testing method. FP is the number of non-disease-models passed the threshold, TN equals the number of non-disease-models failed to pass the threshold.

(3) The **power** is defined as follow

$$\text{power}=\frac{\#(S)}{\#T}$$ (9)

where #*T* denotes the number of datasets that are generated by the same model parameters (#*T* = 100 in our experiment), #(S) is the number of datasets in which the true disease-causing models are found and passed the corresponding evaluation criteria among all #*T* datasets. The power of screening stage (**1**^**st**^ **power**) denotes the rate that the true disease models have been put into the candidate set in 1^st^ stage. The power of testing stage (**2**^**nd**^ **power**) is the rate that the true disease models have been passed the significant threshold of G-test, which is equal to TP/#*T*.

### Simulation datasets

#### Model-based data

We perform experiments on 82 simulated data sets to investigate the performance of FHSA-SED algorithm. These data sets are divided into two categories: disease loci with main effects (DME 1- DME 12) and disease loci without main effects (**DNME 1** –**DNME** 70).

(1) **Simulation 1** (disease loci with main effects**: DME).**

The DME model has both main effects and interaction effects. Twelve disease models (Model 1-Model 12) [4], which are composed of multiplicative model, threshold model and concrete model, are adopted in Simulation 1.

**DME 1- DME 4** (H^2^ = 0.005, MAF = 0.05, 0.1, 0.2 and 0.5) are multiplicative models with two disease locus, in which the disease prevalence given the frequency of genotype combination increases multiplicatively with the incremental presence of the disease. The genetic heritability (H^2^) of DME 1- DME 4 are all equal to 0.005, minor allele frequencies (MAF) of them equal 0.05, 0.1, 0.2 and 0.5, respectively.

It is very difficult to identify the disease locus from the four DME models due to having very low genetic heritability. The fitness landscape of DME 1 is shown in [**Fig E1 in S3 File]**, in which the fitness value of disease-causing SNP-pair is more or less similar to those of some non-pathogenic SNP-pairs. As seen from [**Fig E1 in S3 File]** that the disease-causing SNP-pair (10, 80) has not very significant difference with some other two-locus models, which makes the search algorithm easy to be deviated from correct direction and leads to the miss of the disease-causing two-locus model.

**DME 5- DME 8** (H^2^ = 0.02, MAF = 0.05, 0.1, 0.2 and 0.5) are the threshold models in which the prevalence of genotype frequency does not increase until the number of disease alleles pass the threshold). [**Fig E2 in S3 File]** is the fitness landscape of DME 8, which has strong marginal effect and interaction effect. From [**Fig E2 in S3 File]**, a SNP marker with strong marginal effect (e.g. SNP marker 10 and SNP marker 80) would form many false disease models with other SNP markers that are not truly associated with the phenotype state.

**DME 9- DME 12** (H^2^ = 0.02, MAF = 0.05, 0.1, 0.2 and 0.5) are the concrete model [49]. [**Fig E3 in S3 File]** is the fitness landscape of DME 12, which shows the model with low marginal effect and strong interaction effect. It can be seen from [**Fig E3 in S3 File]** that a SNP-pair with very weak marginal effect is just like an isolated point.

In Simulation 1, the parameters and the values of penetrance of 12 models are given in [**Table E-1 in S3 File]**. The corresponding data sets are generated using the software GAMETES_2.0 [50]. The disease loci of all generated datasets with GAMETES_2.0 are on the last two SNP markers. In order to avoid position preference for an optimization algorithm, we exchange the places of disease locus to other positions randomly. In each data set, 100 SNPs and 1000 SNPs are respectively simulated.

(2) **Simulation 2** (**disease loci with no main effects: DNME)**

The DNME model only has the interaction effects without the marginal effects. We adopt 70 epistatic models which have different genetic heritability H^2^ (0.01, 0.025, 0.05, 0.1, 0.2, 0.3 and 0.4), MAF (0.2 and 0.4) and different penetrance values. The data corresponding to the 70 models was downloaded from http://discovery.dartmouth.edu/epistatic_data [51]**.** These data sets have 1000 attributes, the first two being functional, the remainder randomly generated. [**Fig E4 in S3 File]** is the fitness landscape of a DNME model (MAF = 0.4, H^2^ = 0.025). It can be seen from [**Fig E4 in S3 File]**, the disease-causing SNP-pair is almost an isolated point without any associated neighborhood that would make the heuristic search algorithm difficultly to find the veritable disease model. The penetrance Tables of 70 DNME models are provided in [**Table E-2 in S3 File].**

#### Results comparison and analysis on model-based data

**In Simulation 1,** we compare FHSA-SED algorithm with MACOED and CSE.

**Fig 4:**
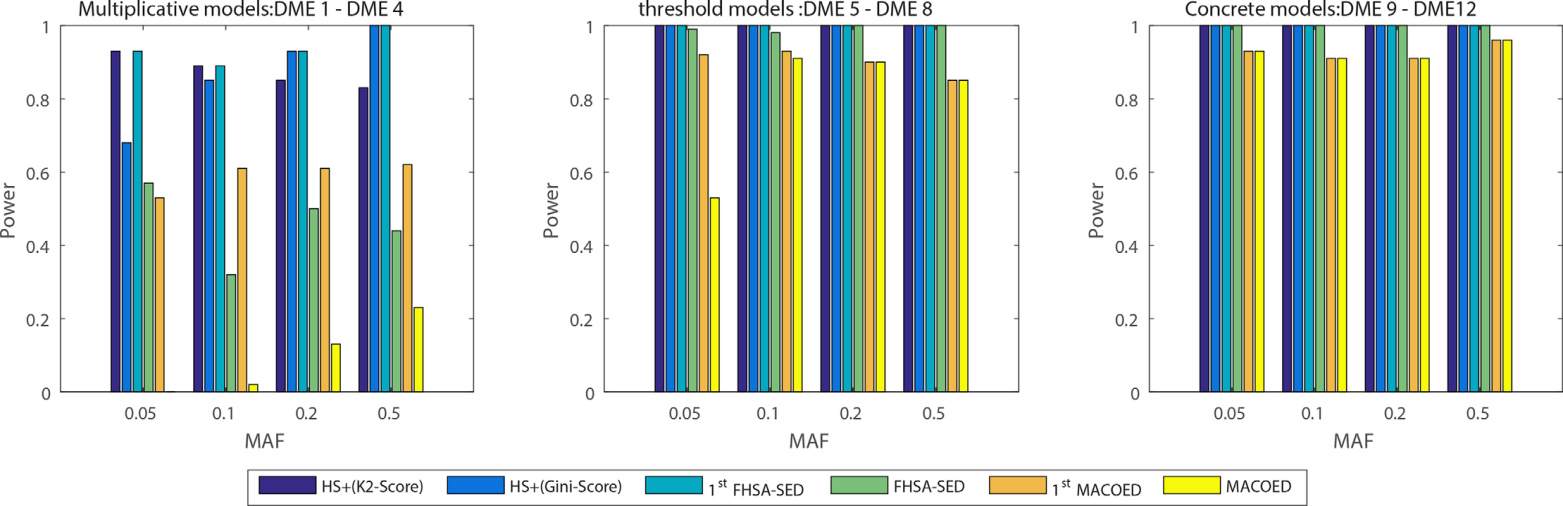
The power comparison on three DME models: (a) The left figure is the multiplicative model (H2 = 0.005); (b) The middle figure is threshold model (H2 = 0.02); (c) The right figure is the concrete model (H2 = 0.02).

**Fig 5:**
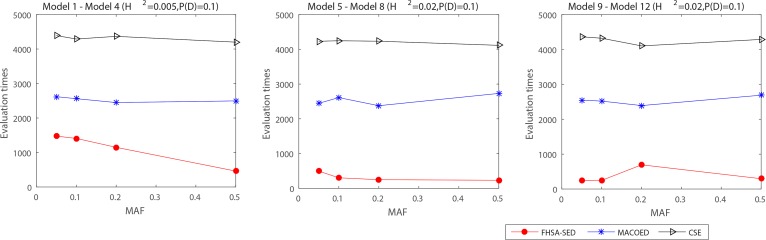
The evaluation times on DME1 -DME 12 for three algorithms: FHSA-SED, MACOED and CSE. (1) The left figure illustrate the evaluation time of three algorithm on DME 1~DME 4 (H^2^ = 0.005, P(D) = 0.1).(2) The middle figure presents the evaluation time of three algorithm on DME 5~DME 8 (H^2^ = 0.02, P(D) = 0.1).(3) The right figure presents the evaluation time of three algorithm on DME 9~DME 12 (H^2^ = 0.02, P(D) = 0.1).

**Fig 6:**
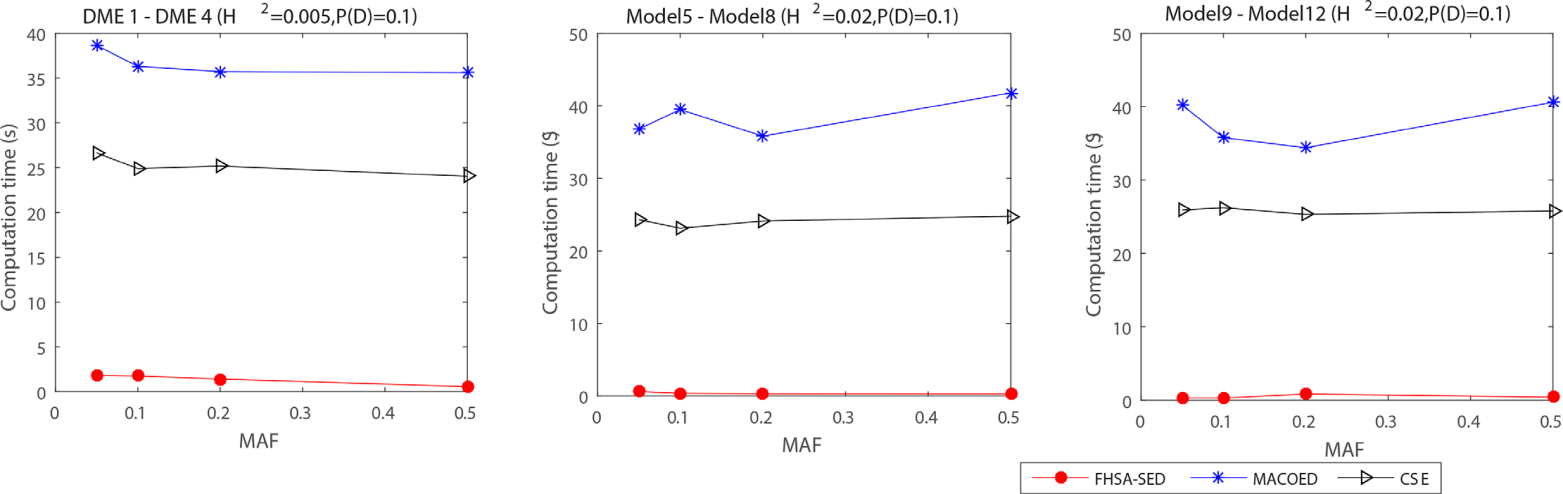
The computation time on DME1 -DME 12 for three algorithms: FHSA-SED, MACOED and CSE. (1) The left figure illustrate the computation time (s) of three algorithm on DME 1~DME 4 (H^2^ = 0.005, P(D) = 0.1).(2) The middle figure presents the computation time (s) of three algorithm on DME 5~DME 8 (H^2^ = 0.02, P(D) = 0.1).(3) The right figure presents the computation time (s) of three algorithm on DME 9~DME 12 (H^2^ = 0.02, P(D) = 0.1).

**Fig 7:**
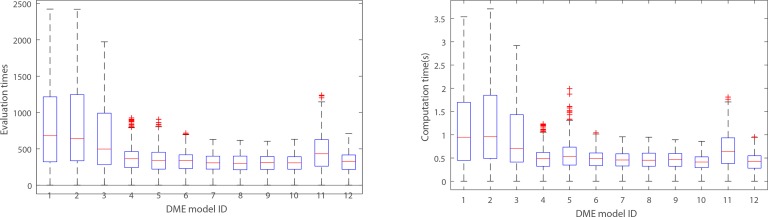
The statistical box plots of FHSA-SED algorithm. Illustrating the distribution of evaluation times and computation time (s). (1) The left figure illustrates the statistical distribution of evaluation times for 12 DME models for 100*5 datasets (100 datasets for each model, and FHSA-SED runs 5 times repeatedly for each data set). (2) The right figure illustrates the corresponding statistical distribution of computation times.

**Fig 8:**
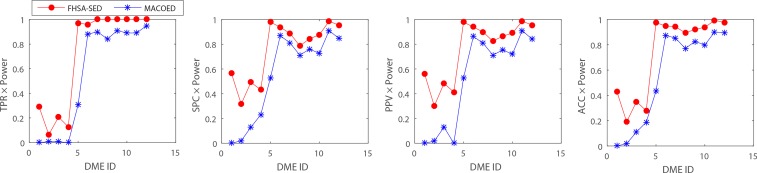
The performance (TPR, SPC, PPV and ACC) on DME1 -DME 12 for FHSA-SED and MACOED algorithms. TPR, SPC, PPV and ACC, which are all multiplied by the corresponding power of each algorithm for 12 DME models, are shown in four sub figures in Fig 8.

**Figs 4–7**present the power, evaluation times and computation time of three algorithms on 12 DME models for the datasets which have 100 SNP markers and quantitative comparisons are also presented in Table 2. In order to further evaluate the performance of FHSA-SED algorithm, we compared four performance metrics (**TPR, SPC, PPV and ACC**) of FHSA-SED and MACOED algorithms on the DME models. Our results are presented in **Fig** 8 and Table 3.

**Table 2:** Powers, evaluation times and computation time for FHSA-SED, MACOED and CSE algorithms (100 SNP markers).

	power							Mean evaluation Times			computation time		
Model	HS+(K2-Score)	HS+(Gini-Score)	1^st^ FHSA-SED	FHSA-SED	1^st^ MACOED	MACOED	CSE	FHSA-SED	MACOED	CSE	FHSA-SED	MACOED	CSE
DME-1	93%	68%	93%	57%	53%	0%	16%	1475.00	2618.33	4389.9	1.82	38.64	26.65
DME-2	89%	85%	89%	32%	61%	2%	18%	1408.72	2560.04	4290.9	1.74	36.31	24.90
DME-3	85%	93%	93%	50%	61%	13%	18%	1149.08	2448.03	4367.4	1.41	35.73	25.18
DME-4	83%	100%	100%	44%	62%	23%	22%	460.65	2495.70	4200.0	0.58	35.60	24.04
DME-5	100%	100%	100%	99%	92%	53%	20%	495.77	2446.50	4232.0	0.61	36.80	24.30
DME-6	100%	100%	100%	98%	93%	91%	21%	303.11	2610.45	4245.9	0.37	39.48	23.14
DME-7	100%	100%	100%	100%	90%	90%	23%	248.43	2377.67	4233.3	0.30	35.82	24.11
DME-8	100%	100%	100%	100%	85%	85%	30%	232.16	2731.15	4114.5	0.29	41.78	24.80
DME-9	100%	100%	100%	100%	93%	93%	16%	241.46	2548.91	4362.0	0.30	40.20	25.91
DME-10	100%	100%	100%	100%	91%	91%	23%	247.45	2519.15	4321.5	0.30	35.77	26.22
DME-11	100%	100%	100%	100%	91%	91%	28%	693.27	2391.45	4101.9	0.85	34.40	25.32
DME-12	100%	100%	100%	100%	96%	96%	19%	295.48	2689.01	4287.3	0.42	40.61	25.78

**Table 3:** The performance (TPR, SPC, PPV, FDR, ACC) comparisons for FHSA-SED and MACOED (100 SNP markers).

Model	FHSA-SED					MACOED				
	power	TPR	SPC	PPV	ACC	power	TPR	SPC	PPV	ACC
**DME-1**	57%	51.16%	99.28%	98.61%	75.22%	0%	0.00%	100.00%	0.00%	81.56%
**DME-2**	32%	20.27%	98.76%	94.23%	59.51%	2%	4.55%	100.00%	100.00%	85.00%
**DME-3**	50%	41.34%	98.63%	96.80%	69.99%	13%	33.33%	99.20%	85.71%	90.91%
**DME-4**	44%	29.00%	98.12%	93.91%	63.56%	23%	68.75%	94.98%	62.86%	92.10%
**DME-5**	99%	98.00%	98.87%	98.86%	98.43%	53%	57.78%	100.00%	100.00%	81.82%
**DME-6**	98%	98.00%	95.75%	95.84%	96.87%	91%	96.81%	95.50%	94.79%	96.10%
**DME-7**	100%	100.00%	88.73%	89.87%	94.36%	90%	100.00%	90.09%	89.81%	94.71%
**DME-8**	100%	100.00%	78.72%	82.46%	89.36%	85%	98.94%	83.78%	83.78%	90.73%
**DME-9**	100%	100.00%	84.42%	86.52%	92.21%	93%	97.83%	81.74%	81.08%	88.89%
**DME-10**	100%	100.00%	87.63%	88.99%	93.81%	91%	97.85%	79.83%	79.13%	87.74%
**DME-11**	100%	100.00%	98.63%	98.65%	99.32%	91%	97.83%	100.00%	100.00%	99.06%
**DME-12**	100%	100.00%	95.36%	95.57%	97.68%	96%	98.94%	88.29%	87.74%	93.17%

In **Fig** 4 and **Table** 2, the powers of HS+ (K2-Score), HS+ (Gini-Score) and 1^st^ FHSA-SED are respectively equal to $\frac{\#S_{1}^{1st}}{\#\text{T}}$, $\frac{\#S_{2}^{1st}}{\#\text{T}}$ and $\frac{\#S_{}^{1st}}{\#\text{T}}$, where #*T* denotes the number of datasets that are generated by the same parameters (#*T* = 100 in our experiment), $\#S_{1}^{1st}$, $\#S_{2}^{1st}$ and #*S*^1*st*^ denote the numbers of the true two-locus disease models (in #T simulate datasets) having been put into HM1, HM2 and HM, respectively. Likewise, 1^st^ MACOED is the union power of ACO+(K2-Score and ACI-Score) in the screening stage. The powers of FHSA-SED and MACOED are the rate that the true disease models have been passed the significant threshold *P-value* of G-test and Chi-square test respectively.

It is indicated from **Fig** 4 and Table 2 that the FHSA-SED algorithm outperforms MACOED and CSE methods on all 12 DME models, in which the HS algorithm with K2 Scoring criterion (HS+K2-Score) has a higher power than HS+(Gini-Score) on DME 1–2, however, HS+(Gini-Score) is more powerful on DME 3 and DME 4 than HS+(K2-Score) algorithm. This illustrates that the two scoring criterions in 1^st^ FHSA-SED can complement each other. We can found from column 4 (1^st^ FHSA-SED) and column 5 (FHSA-SED) in Table 2 that the power of FHSA-SED, for DME 1 ~ DME 4, is lower than that of 1^st^ FHSA-SED because part of shortlisted candidates of 1^st^ FHSA-SED failed to pass the significant threshold of G-test, resulting in type II errors. Which because, for DME 1 ~ DME 4, there are very small significant difference between case data and control data. If the significant threshold of G-test is relaxed, some false disease models might pass the significant threshold for DME 7 ~ DME 12 (type I errors), which error is generally even less acceptable. Nevertheless, the shortlisted candidates in 1^st^ FHSA-SED that have failed to pass the significant threshold of G-test are worth studying further by employing or developing effective approaches.

As is illustrated in **Fig** 5, **Fig 6, Fig 7**and **Table 2**, the evaluation times and the computation time of our method are significantly less than other two methods. For three type of DME models (multiplicative model: DME 1–4, threshold model: DME 5–8 and concrete model: DME 9–12), the mean evaluation times of our method are less than 1500, 300 and 600, respectively, and the mean computation time is less than 2s, 0.6s and 1s. **Fig** 7 presents the statistical box plots of FHSA-SED about evaluation times and computation time (100*5 datasets are used to test for each DME model). It can be seen from Table 2 that FHSA-SED algorithm only takes a very small amount of evaluation times and spend very little computation time for most of the datasets, for which the exhaustive search algorithm requires 4950 (100*99/2) evaluation times using K2-scoring and Gini-scoring criterions respectively and takes approximate 5.2s for each dataset with 100 SNP markers. This illustrates that the FHSA-SED can effectively reduce the evaluation times and decrease the computation time in solving DME models. However, we find out that MACOED and CSE take more the computation time than exhaustive search algorithm on DME models with 100 SNP markers, which demonstrates that MACOED and CSE algorithms themselves are more time-consuming than FHSA-SED in the process of search.

A detailed view of Table 3 shows that the TPR of FHSA-SED on most of DME models is larger than that of MACOED. Yet the TPR value on DME-4 is relatively smaller than that of MACOED, which is because some SNP-pairs that have been obtained in the 1^st^ FHSA-SED are rejected in testing stage (*see the Table 2, the powers of 1*^*st*^ FHSA-SED *on DME 1~DME 4 are equal to 93%*, *89%*, *93% and 100%*, *which are much larger than powers of 1*^*st*^ *MACOED*), which makes the false negative rate a little high because the significantly difference (in multiplicative models: DME 1~ 4) between case data and control data is not very obvious. However, on DME 5 ~ DME 12, the TPR, SPC, PPV and ACC of FHSA-SED algorithm are all better than or not significant differences with those of MACOED.

As can be noticed in Table 3, for some models, the values of TPR are larger than or equal to the values of power, which because some disease models have not been found in the 1^st^ screening stage. For example, if in the 1^st^ FHSA-SED, 55 true disease models have been found from 100 datasets (1^st^ power = 55%), and 2 models among these 55 disease models failed to pass the threshold of G-test in 2^nd^ FHSA-SED (TP = 53, FN = 2), then TPR = TP / (TP + FN) = 96%, power = 53%, and TPR>power.

As also can be found in Table 3, the CSE algorithm has not been contained, which because the goal of CSE is to find the disease models using Cuckoo search algorithm and Bayesian evaluation criterion. For each simulation dataset, the output of CSE is Yes (if the only disease-causing has been found) or No (if the disease-model has not been found), and CSE does not contain any statistical analysis for the output results. Therefore, to be fair, the TPR, SPC, PPV and ACC are not included in CSE algorithm.

In order to make a fair comparison, we multiply TPR, SPC, PPV and ACC by corresponding power of each algorithm for each model. Results are presented in **Fig 8**, indicating that our method is more effective than MACOED on all 12 DME models.

**Fig 9:**
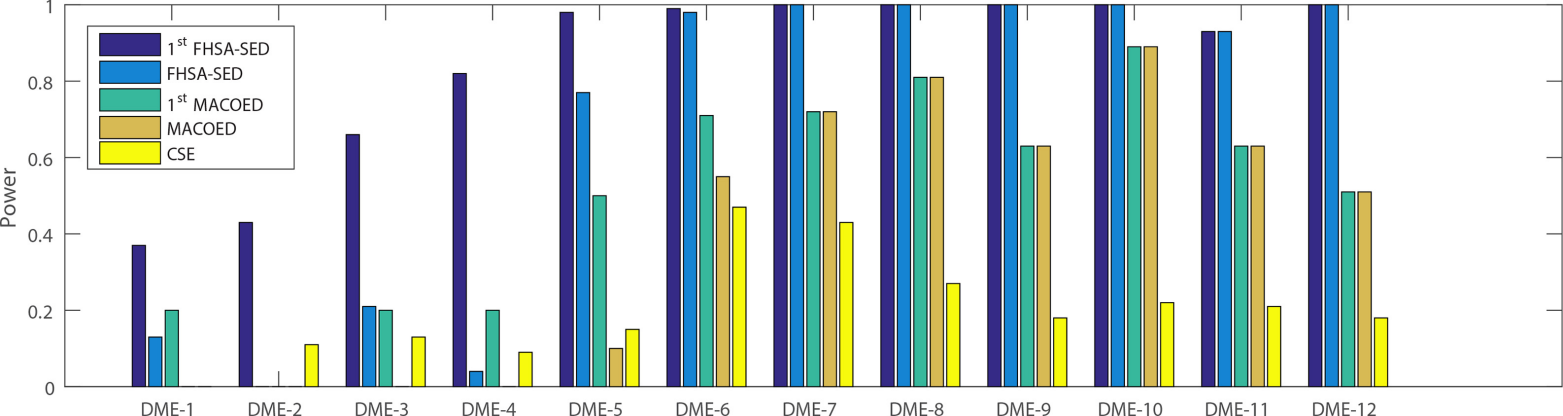
The power comparison on 12 DME models with 1000 SNP markers.

**Fig 10:**
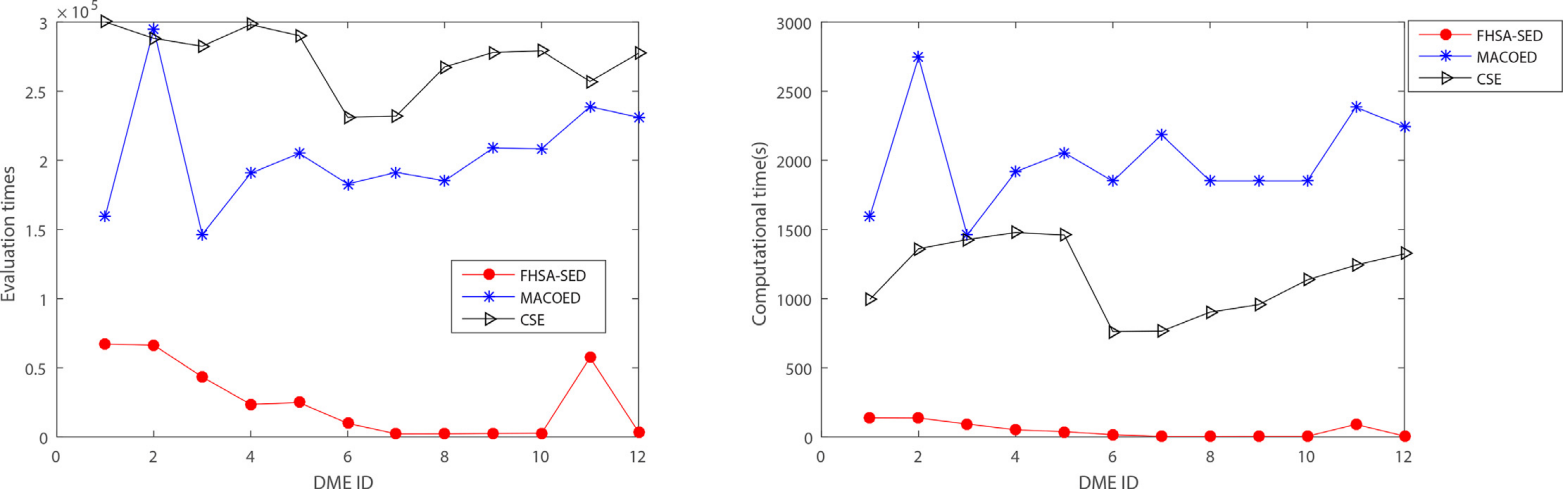
The computation time and evaluation times on 12 DME models with 1000 SNP markers.

We also perform our algorithm on 12 DME models for datasets which have 1000 SNP markers; **Fig 9 and Fig 10**present the power, evaluation times and computation time of three algorithms on 12 DME models and quantitative comparisons are presented in Table 4 and Table 5.

**Table 4:** Powers, evaluation times and computation time for FHSA-SED, MACOED and CSE algorithms (1000 SNP markers).

	power							Mean evaluation Times			computation time (s)		
Model	HS+(K2-Score)	HS+(Gini-Score)	1^st^ FHSA-SED	FHSA-SED	1^st^ MACOED	MACOED	CSE	FHSA-SED	MACOED	CSE	FHSA-SED	MACOED	CSE
DME-1	37%	25%	37%	13%	20%	0%	0%	67088.1	159627	300000	137.3	1598	995
DME-2	42%	39%	43%	0%	0%	0%	11%	66366.9	294749	288150	135.8	2744	1360
DME-3	56%	66%	66%	21%	20%	0%	13%	43360.8	146310	282450	93.7	1460	1427
DME-4	56%	82%	82%	4%	20%	0%	9%	23487.6	190546	298100	50.1	1917	1477
DME-5	98%	98%	98%	77%	50%	10%	15%	24765.8	205019	290100	37.2	2055	1459
DME-6	99%	99%	99%	98%	71%	55%	47%	9811.6	182828	231100	15.3	1848	761
DME-7	100%	100%	100%	100%	72%	72%	43%	2122.4	191243	231900	3.1	2184	764
DME-8	100%	100%	100%	100%	81%	81%	27%	2171.6	185136	267450	3.1	1850	902
DME-9	100%	100%	100%	100%	63%	63%	18%	2366.8	208807	277950	3.7	1850	956
DME-10	100%	100%	100%	100%	89%	89%	22%	2559.5	208357	279150	3.7	1850	1135
DME-11	93%	93%	93%	93%	63%	63%	21%	57318.2	238678	256950	89.9	2383	1242
DME-12	100%	100%	100%	100%	51%	51%	18%	3602.1	231101	277350	5.2	2243	1324

**Table 5:** The performance (TPR, SPC, PPV, FDR, ACC) comparisons for FHSA-SED and MACOED (1000 SNP markers).

Model	FHSA-SED						MACOED					
	power	TPR	power×TPR	SPC	PPV	ACC	power	TPR	power×TPR	SPC	PPV	ACC
**DME-1**	13%	35%	5%	98%	95%	98%	0%	0%	0%	100%	0	100%
**DME-2**	0%	0%	0%	99%	0%	98%	0%	0%	0%	100%	0	100%
**DME-3**	21%	32%	7%	98%	95%	98%	0%	0%	0%	97%	0%	97%
**DME-4**	4%	5%	0%	98%	74%	98%	0%	0%	0%	98%	0%	98%
**DME-5**	77%	79%	61%	100%	100%	100%	10%	100%	10%	100%	100%	100%
**DME-6**	98%	99%	97%	98%	98%	98%	55%	100%	55%	89%	71%	91%
**DME-7**	100%	100%	100%	92%	93%	92%	72%	100%	72%	50%	63%	73%
**DME-8**	100%	100%	100%	71%	77%	71%	81%	100%	81%	0%	67%	67%
**DME-9**	100%	100%	100%	82%	85%	83%	63%	100%	63%	59%	46%	70%
**DME-10**	100%	100%	100%	87%	89%	87%	89%	100%	89%	100%	100%	100%
**DME-11**	93%	100%	93%	99%	99%	99%	63%	100%	63%	100%	100%	100%
**DME-12**	100%	100%	100%	97%	97%	97%	51%	100%	51%	92%	83%	94%

As shown in **Fig** 9, **Fig 10**and Table 4, the FHSA-SED has much higher power than MACOED and CSE for most of DME models, and the evaluation times and runtime of FHSA-SED are also far less than those of MACOED and CSE. We can found from Table 5 that, for some models (e.g. DME 5 and DME 6), the TPR of MACOED is higher than that of FHSA-SED, which because only part of disease-models with significant difference between case and control have been discovered in 1^st^ stage of MACOED, but many other unobvious disease-models that have low significant difference between case data and control data have not been found. However, if we think about the value of power, the power×TPR in FHSA-SED is much higher than that in MACOED, which illustrates that the FHSA-SED is more powerful in detecting various disease-models than MACOED.

**Fig 11:**
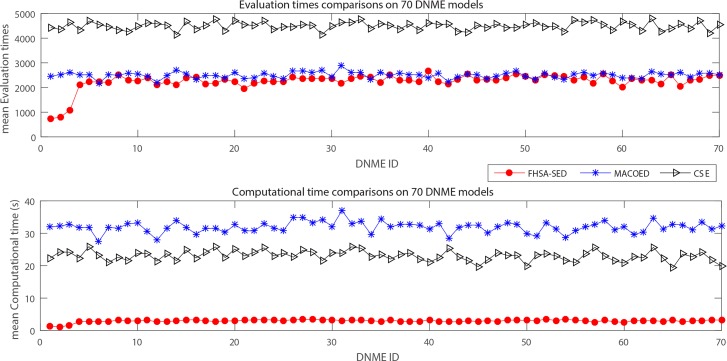
The evaluation times and computation time on 70 DNME models for three algorithms (100SNP markers).

**In Simulation 2**, performance comparisons on 70 DNME models are performed. [**Fig E5-E6 in S3 File]** display the powers of three algorithms, **Fig 11**and [**Table E**-3 **in S3 File]** present the evaluation times and computation time for three algorithms when the number of SNP markers equals 100. Other four performance metrics (**TPR, SPC, PPV and ACC**) are also shown in [**Table E**-4 **in S3 File]**.

From **Fig** E5 and [**Fig** E6 **in S3 File]**, we can find that the power of 1^st^ FHSA-SED is higher than CSE for all 70 DNME models, and its power is better than that of 1^st^ MACOED for most of DNME models**.** FHSA-SED method has distinct advantages over MACOED and CSE algorithm on power for DNME-1~DNME-5 and DNME-36~DNME-40 (the genetic hereditability H^2^ = 0.01). For other DNME models, the FHSA-SED and MACOED have nearly equal powers.

In addition, **Fig** 11 indicates that the FHSA-SED algorithm takes very less **computation time** than MACOED and CSE. MACOED spends most time among three algorithms, which is almost 10 times what FHSA-SED spends. It is indicated from **Fig** 11 that FHSA-SED requires slightly less evaluation times than MACOED for DNME models.

As shown in [**Table E**-4 **in S3 File]**, the FHSA-SED algorithm has a close performance to the MACOED algorithm for most of DNME models. However, for DNME-2, DNME-36~DNME-40, the TPR of FHSA-SED is lower than that of MACOED, which is because 1^st^ FHSA-SED has much higher power than 1^st^ MACOED (See **Table E**-3 **in S3 File**), and small significant threshold value (*P*-value = 0.01/4950) make some candidate solutions prone to obstructed pass the threshold of *G*-test in the testing stage, which means some true candidate solutions fail to pass the *G*-test due to small significant threshold (*P*-value) although they have successfully passed the screening in 1^st^ FHSA-SED (these candidate solutions maybe filtered out in 1^st^ MACOED), This illustrates that candidate solutions in 1^st^ FHSA-SED are very worth studying further, and which are called for a good testing method that embrace the complex disease models in future work.

In order to investigate the performance of FHSA-SED on solving DNME models, we test it using 70 DNME models which have 1000 SNP markers. The results are illustrated **in [S3 File]** (**Fig** E7 ~ **Fig** E8 and **Table E**-5).

### Experiments on AMD real data

According to the previous analysis for simulation experiments, the proposed algorithm has a good performance on 70 simulation models. In this section, we conduct experiments on a real data set (AMD: Age-related macular degeneration) [52] using our proposed algorithm. The AMD dataset contains 103611 SNPs genotyped for 50 controls and 96 cases. Our goal of the experiment is to find quickly the disease-causing two SNP loci in AMD dataset using FHSA-SED algorithm.

Firstly, SNP loci with p-values from G-test less than 0.3 are removed from AMD dataset. Subsequently, 31341 SNP loci remain in the AMD dataset.

The setting of parameters for FHSA-SED algorithm is as follows:

○ ||HM1|| = 500; ||HM2|| = 500;
○ maximum evaluation times for SNP-pairs is equal to 3E+6;
○ The p-value threshold for SNP-pairs equals 0.05(313412)
○ Other setting of parameters is the same as those of Table 1.

The experiment took 4 hours approximately. There are 638 SNP-pairs (*See* S4 File) survived in the final output set.

**Fig 12:**
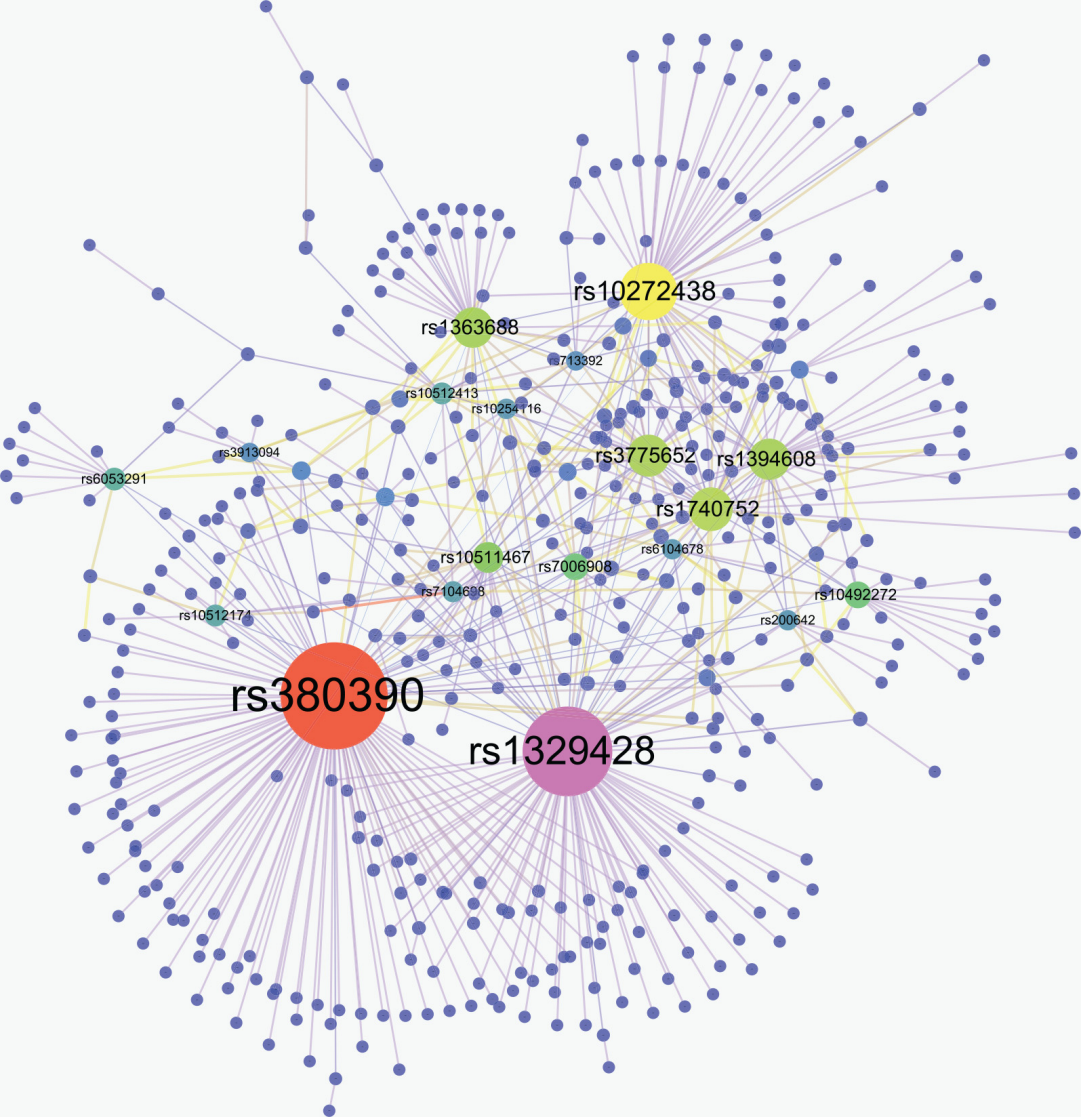
SNP-SNP network. There are 638 SNP-pairs having passed the screening and testing in final results. In Fig 12, a node denotes a SNP locus. Two linked nodes represent one SNP-pair of final 638 SNP-pairs. The larger the node, the more nodes linked with it.

**Fig 13:**
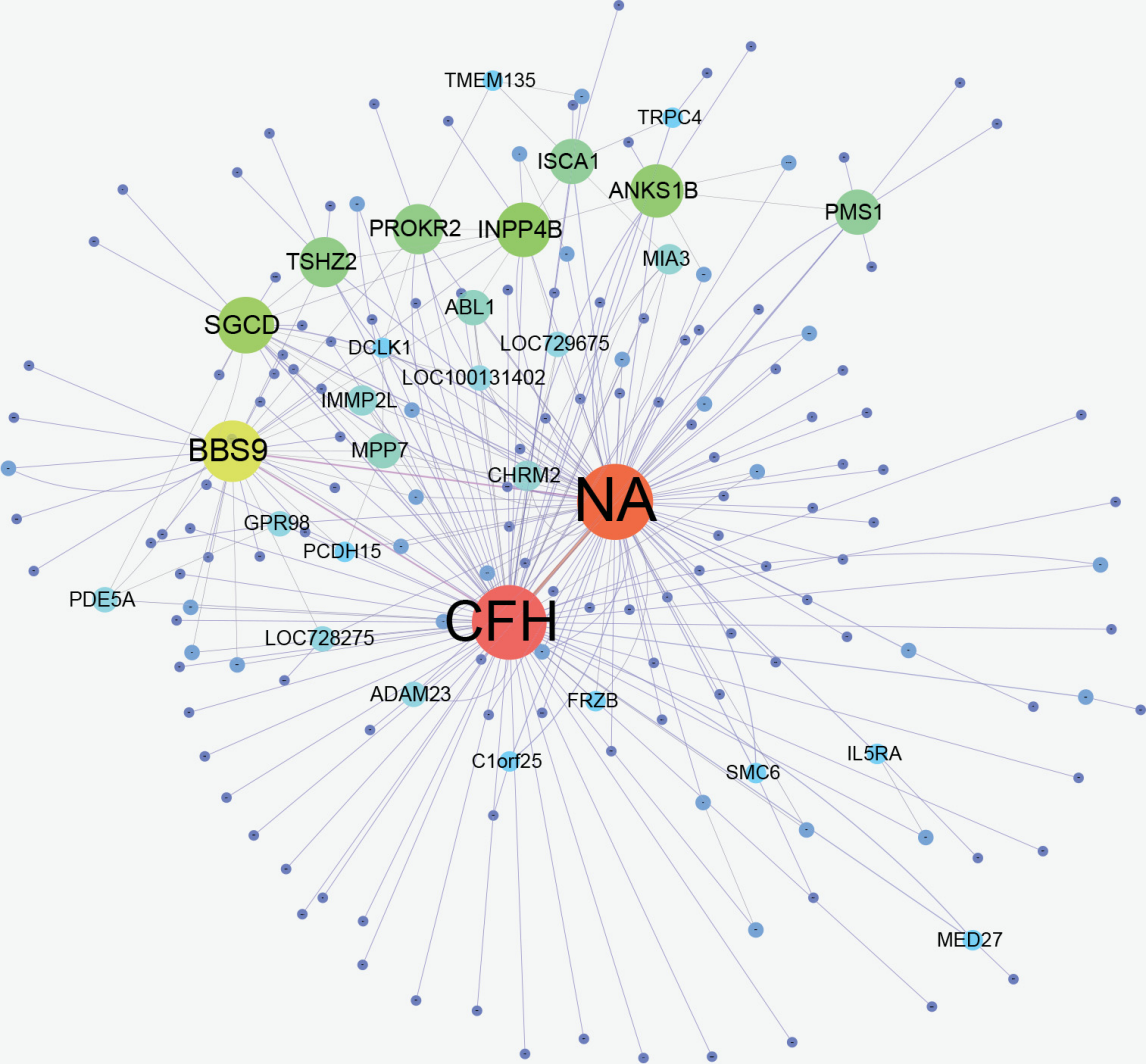
Gene-gene network. The gene in Fig 13 is mapped from SNP, each SNP loci corresponds to a gene. A gene contains one or more SNPs, for example, *'rs380390'* and *'rs1329428'* are all mapped in gene: CFH.

All these 638 SNP-pairs are displayed in Fig 12, and the corresponding gene-pairs (mapped from SNP) are presented in Fig 13. It can be seen evidently from Fig 12 that three SNPs '*rs380390*', '*rs1329428*' and '*rs10272438*' are associated with more other SNPs. In Fig 13, CFH, NA and BBS9 are linked with more other genes, where NA is not a gene, which means many SNPs are not in a gene region.

Similar to literatures [53–54], we select 26 top SNPs whose frequency is larger than 5 in the 638 SNP-pairs. In Table 6, the top two highest frequency SNPs (*'rs380390'* and '*rs1329428*') which are all in an intron gene CFH, have been widely believed to be significantly associated with AMD [54–55]. Eight high frequency SNPs ranked from third to tenth may be also genetic factor contributing to the underlying mechanism of AMD. To our knowledge, *'rs10272438*',*'rs1740752*', '*rs1394608'*, '*rs1363688'*, ' *rs7006908'* and '*rs10492272*' have been reported before; however, '***rs3775652***' and '***rs10511467***' have not been reported before, which need further to be studied and confirmed whether these SNPs are truly associated with AMD by developing a more efficient test method or using large scale samples.

**Table 6:** Top 26 high-frequency SNPs in 638 SNP-pairs on AMD dataset.

Order	SNP	P-value	Chromosome	Gene	Frequency	Reported in ref
**1**	***rs380390***	6.2E-07	1	CFH	121	[23, 28,54–58]
**2**	*rs1329428*	5.99E-06	1	CFH	98	[23, 28,54–58]
**3**	*rs10272438*	9.67E-06	7	BBS9	57	[56]
**4**	*rs1740752*	4E-05	10	NA	40	[54]
**5**	*rs3775652*	3.73E-07	4	INPP4B	38	no reported
**6**	*rs1394608*	4.21E-05	5	SGCD	38	[54,57–58]
**7**	*rs1363688*	3.84E-05	5	NA	36	[28,54]
**8**	***rs10511467***	2.91E-05	9	NA	**24**	no reported
**9**	*rs7006908*	0.000138	8	NA	19	no reported
**10**	*rs10492272*	0.000259	12	ANKS1B	19	[57]
**11**	*rs6053291*	0.000196	20	PROKR2	14	[62]
**12**	*rs10512413*	0.000211	9	ABL1	13	no reported
**13**	*rs10512174*	0.000194	9	ISCA1	13	[28, 54, 58–61]
14	*rs7104698*	0.000159	11	NA	12	[58]
15	*rs6104678*	0.000212	20	NA	11	[58]
16	*rs200642*	0.000368	20	TSHZ2	11	no reported
17	*rs10254116*	0.00014	7	BBS9	11	[23,56,59]
18	*rs713392*	0.001531	7	IMMP2L	10	no reported
19	*rs3915771*	0.000772	5	NA	9	no reported
20	*rs3914244*	1.44E-05	12	NA	9	no reported
21	*rs1233255*	0.000472	2	PMS1	8	[60]
22	*rs10485193*	0.004187	10	NA	8	no reported
23	*rs1930022*	2.37E-06	9	NA	7	no reported
24	*rs10507949*	0.000574	13	NA	7	[28]
25	*rs9294603*	9.7E-05	6	NA	7	no reported
26	*rs206695*	0.001028	6	LOC728275	5	no reported

In Table 7, top-20 SNP-pairs are presented in terms of *P-value* of G-test. It is noted that there are 15 SNP-pairs associated with three SNPs: '*rs380390*', '*rs1329428*' and '*rs10272438*', other five SNP-pairs are associated with two unreported SNPs *'rs3775652'* and *'rs10511467'*.

**Table 7:** Top 20 SNP-pairs in terms of P-value of G-test.

Order of P-Value	SNP1			SNP2			P-VALUE of G-TEST (SNP1-SNP2)
	Name	Index in AMD	P-VALUE	Name	Index in AMD	P-VALUE	
1	***rs380390***	43748	6.20E-07	***rs2224762***	97535	1.99E-02	2.44471E-12
2	***rs380390***	43748	6.20E-07	***rs2402053***	57476	8.05E-03	2.65932E-12
3	***rs380390***	43748	6.20E-07	***rs10512937***	77802	2.52E-03	4.67459E-12
4	***rs380390***	43748	6.20E-07	***rs1926489***	7026	1.09E-01	5.68912E-12
5	***rs380390***	43748	6.20E-07	***rs10497346***	94452	2.96E-01	8.46601E-12
6	***rs380390***	43748	6.20E-07	***rs2380684***	75884	4.56E-02	9.2214E-12
7	***rs1329428***	54108	5.99E-06	***rs9328536***	31604	3.34E-03	2.02139E-11
8	***rs1329428***	54108	5.99E-06	***rs7467596***	79546	3.34E-03	2.02139E-11
9	***rs1329428***	54108	5.99E-06	***rs3775652***	12147	3.73E-07	2.17868E-11
10	***rs3775652***	12147	3.73E-07	***rs725518***	46516	4.87E-05	2.44424E-11
11	***rs380390***	43748	6.20E-07	***rs10483314***	16459	1.89E-03	2.87683E-11
12	***rs380390***	43748	6.20E-07	***rs1363688***	80178	3.84E-05	3.07928E-11
13	***rs10511467***	76784	2.91E-05	***rs1046592***	65049	2.14E-03	3.43886E-11
14	***rs10511467***	76784	2.91E-05	***rs12046095***	68566	2.14E-03	3.43886E-11
15	***rs10511467***	76784	2.91E-05	***rs10489581***	14227	2.14E-03	3.43886E-11
16	***rs10511467***	76784	2.91E-05	***rs10502376***	22505	2.14E-03	3.43886E-11
17	***rs380390***	43748	6.20E-07	***rs10511145***	18229	1.64E-02	3.57226E-11
18	***rs10272438***	33990	9.67E-06	***rs1510134***	82857	7.78E-04	3.86354E-11
19	***rs1329428***	54108	5.99E-06	***rs356054***	44601	6.61E-02	3.95542E-11
20	***rs380390***	43748	6.20E-07	***rs724972***	76613	9.95E-03	4.03304E-11

## Discussion

### Relationship between FHSA-SED and MACOED

In this study, we proposed a HS algorithm using Screening and Testing to identify the SNP-pair **disease** models among all SNP-pairs, which has nearly the same algorithmic framework as MACOED. The key differences lie between FHSA-SED and MACOED:

1. MACOED employs two scoring functions (Bayesian network-based K2-score and logical regression-based AIC-score) to screen the disease models in the first stage, in which logic "and" operation is carried out between the two scoring functions. FHSA-SED also adopts two scoring criteria (K2-score and Gini-score) to evaluate the association of two-locus models with disease status, and the logic "or" operation is performed between two scoring criteria. Therefore, in the screening stage, the MACOED algorithm adopts stricter criteria to screen the disease models than FHSA-SED algorithm; however, MACOED will make some true disease models be filtered out.
2. MACOED is intended to search disease models via ACO algorithm (employing large population size). In FHSA-SED, HS algorithm is employed to detect disease-causing SNP-pairs and a local search algorithm is presented to discover no-visited solutions in constant time. In MACOED, logical regression-based AIC-score requires some iteration to calculate regression coefficients, which take much more time than the Gini-scoring in FHSA-SED.
3. In MACOED, Pearson's *χ*^2^ test is performed on the no-dominant solutions obtained in the screening stage. FHSA-SED employs the G-test to test the candidate solutions in the testing stage.

We investigate the performance of FHSA-SED algorithm via three simulation experiments:

1. 12 DME models: the disease loci have both main effects and interaction effects.
2. 70 DNME models: the disease loci have only the interaction effects without the main effects.
3. AMD dataset that contains 103611 SNPs genotyped for 50 controls and 96 cases.

Results of DME models indicate that FHSA-SED is more effective in seven performance metrics than MACOED and CSE, especially, it takes very fewer evaluation times of SNP-pairs and much less computation time than MACOED.

The simulation experiment on DNME models demonstrates that the performances of our method on power, evaluation times, TPR, SPC, PPV, and ACC are better than or equivalent to those of MACOED, and computation time of our method is much less than that of MACOED.

The real data AMD experiment also indicates that our method has found out the known disease loci successfully and also discovered some new suspected disease loci.

### Advantages and Limitations of FHSA-SED

#### Advantages

FHSA-SED is a fast swarm intelligent optimization algorithm and is a model-free method that assumes neither any prior distribution nor any particular disease models. Two scoring functions in FHSA-SED can complement with each other and enhance the detection power of the two-locus disease models. Our algorithm detects the two-locus disease models without evaluating all genotype combinations by using a tabu table, and it can achieve the search performance of exhaustive search algorithm when maximum model evaluation times (MMs) is equal to the number of genotype combinations. So it is a global optimization algorithm for the detection of two-locus disease models. FHSA-SED can be easily implemented using parallel computing via splitting the tabu table (TT) into some small tabu table and each computing can be performed independently in a small tabu table.

#### Limitations

FHSA-SED consumes much large memory due to the considerable size of tabu table (TT). The current version of FHSA-SED cannot deal with the detection of multi-SNPs (>2) disease models. Facing various type of disease models, the balance between type I errors and type II errors has not yet to be satisfactorily solved. For the DME models with small genetic heritability H2 or minor allele frequency (MAF), the type II errors might occur, and for the models with strong marginal effects, the type I errors might be generated.

#### Future work

To our knowledge, there does not exist a very powerful approach in detecting high-order disease models at GWAS, therefore, at this moment, multi-loci interaction detection have many room to explore. In addition, powerful identification algorithms and statistical methods are very needed for high-order disease models. We are also developing a fast niche harmony search algorithm with small size of tabu table for detecting high-order disease models.

## Supporting Information

S1 File The method for Bayesian network scoring criteria and Gini scoring criteria. Including the detail description of Bayesian network scoring and Gini index criteria.
(DOC)

S2 File Standard Harmony algorithm. Including the introduction of standard Harmony algorithm and the flow chart of FHSA-SED algorithm. (DOC)

S3 File The experiments results. All the supplementary experiment data, experiment results and figures.
(DOC)

S4 File 638 SNP-pairs having strongest association with AMD. All 638 SNP-pairs that passed the Screening in 1^st^ stage and the Testing in 2^nd^ stage.
(XLS)

S5 File Matlab source code of FHAS-SED algorithm. (ZIP)
