# Supplementary material for: FHSA-SED: Two-Locus Model Detection for Genome-Wide Association Study with Harmony Search Algorithm
Source: PLoS One. 2016 Mar 25;11(3):e0150669. doi: 10.1371/journal.pone.0150669 (PMC4807955; doi:10.1371/journal.pone.0150669)
Supplement: S1 File — Including the detail description of Bayesian network scoring and Gini index criteria. (DOC) [file pone.0150669.s001.doc]

**Bayesian network scoring criteria and Gini scoring criteria**

**1. K2-Score**

**1.1 Bayesian model**

A Bayesian network (BN) is a type of statistical model (probabilistic directed acyclic graphical model) which represents a set of random variables and their conditional dependencies by using a directed acyclic graph (DAG) ( http://en.wikipedia.org/wiki/Bayesian_network ). In the DAG, Nodes denote random variables, and the edges represent conditional dependences between two linked nodes.

There are more than ten kinds of BN models （*Zhang, Y., & Liu, J. S. 2007；Jiang, X et.al. 2011; Visweswaran S et.al. 2009* ）that have been developed to find causal relationships, perform explanatory analysis ,describe the causal influence and make predictions. In GWAS studies, BN model is also used to detect the interaction effect among SNP markers, which can represent the relationship between genetic variants and disease status.

Let a set of SNP variables $X=\left\{x_{1},x_{2},\cdots,x_{N}\right\}$indicate *N* SNP markers for *S* individuals, phenotype variable be $Y$ with values $\left(y_{1},y_{2},\cdots,y_{J}\right)$ ; we represent the homozygous major allele, heterozygous allele and homozygous minor allele as 0, 1 and 2. In a DAG of Bayesian network for representing the relationships of SNP markers and disease states, there are only directed edges linking from the SNP markers to diseases status, and there is no any edge linked from disease state to SNP markers and no edge connected among SNP markers. In the DAG, if and only if SNP$x_{i}$ is a direct cause of phenotype$y_{j}$, there is a directed edge linking from node$x_{i}$to node$y_{j}$. Figure A1 shows an example of *k-*SNPs epistasis Bayesian network model associated with phenotype$y_{j}$, where $c_{i}=(x_{i_{1}},x_{i_{2}},\cdots,x_{i_{k}})$ is a *k*-combination of genotypes (*k* SNPs markers), $y_{j}(0\leq j\leq J)$denotes the phenotype state.

*...*

*y*_j_

Fig A1 *k-*SNPs epistasis Bayesian network model

A *k*-combinatorial model associated with phenotype can be expressed by the probability distribution (Visweswaran, S., Wong, A. K. I., & Barmada, M. M. 2009) as follow:

$P\left(Y|c_{i}\right)=P(Y|x_{i_{1}},x_{i_{2}},\cdots,x_{i_{k}})$ S2 (1)

where, *Y* is the state variable of phenotype and ($x_{_{i1}},x_{i_{2},\cdots},x_{i_{k}}$ ) a *k*-combination of genotype variables. For a *k*-SNPs combinational model associated with phenotype, there are $k^{3}$ possible genotypic combinations for diploid genomes (each SNP markers has three values: 0, 1 and 2) and each genotypic combination is associated with a probability distribution over the phenotype variable *Y*.

There have been several Bayesian network structure learning models successfully used to identify the SNP interaction, such as score-based methods used to evaluate the association between genotypes and phenotype by scoring the model’s fitness to the data.

**1.2 Bayesian Scoring (K2-Score)**

The Bayesian theorem can be used to evaluate the fitness of any given combinatorial model. Let a sample dataset *D* that contains values for SNPs and phenotype states, the BN model *M* can be expressed as the following equation:

$P(M|D)=\frac{P(D|M)P(M)}{P(D)}$ S2(2)

where, $P(M|D)$ is the posterior probability given sample dataset *D*,$P(M)$is the prior probability of *M*, $P(D|M)$denotes the marginal likelihood. *P(D)* represents the probability of the data *D* , which is a constant for all Bayesian network models based on data set *D*. (Visweswaran, S., Wong, A. K. I., & Barmada, M. M. 2009).). Thus, the equation S2 (2) can be written as

$P(M|D)\propto P(D|M)P(M)$ S2 (3)

where, the symbol "" denotes that the $P(M|D)$ is proportional to $P(D|M)P(M)$.

$P(D|M)$ can be written as follow,

$P(D|M)=\int_{\theta_{M}} P(D|M,\theta_{M})P(\theta_{M}|M)d\theta_{M}$ S2(4)

where, $\theta_{M}$ denotes the parameters of the probability distributions of phenotype variable *Y* given the SNP variables $\left\{x_{1},x_{2},\cdots,x_{N}\right\}$.

According to the theorem 1 given in (*Cooper GF, Herskovits E ,1992),* under the following assumptions:

(1) The values of the phenotype variable are generated via independent identically distributed (i.i.d) sampling from P(*Y |X*).

(2) Prior belief about the distribution *P(Y|X=x_i_)* is independent of prior belief about *P(Y|X=x_j_*) for all values *x_i_* and *x_j_* of *X (x_i_≠x_j_)*.

(3) For all values *x_j_*, prior belief about the distribution *P(Y|X=x_j_*) is modeled using Dirichlet dirtribution with hyperparameters $\alpha_{i}$and $\alpha_{ij}$.

The equation S2(4) has a closed-form solution which is as following expression:

$P(D|M)=\prod_{i=1}^{I} \left(\frac{\Gamma(\alpha_{i})}{\Gamma(n_{i}+\alpha_{i})}\prod_{j=1}^{J} \frac{\Gamma(n_{ij}+\alpha_{ij})}{\Gamma(\alpha_{ij})}\right)$ S2(5)

where, *I* is the number of genotype combinations (there are 3*^k^* SNP markers' combinations if *k*-SNP nodes are connected to the phenotype y_j_) , *J* is the number of phenotype states *Y* ( which is equal to two for case-control dataset). $\Gamma$is the gamma function, $n_{i}$ is the number of cases in the dataset with SNP nodes taking the *i-*th genotype combination, $n_{ij}$ represents the number of cases that belong to the phenotype state *j* where the *k*-SNPs variables have *i*-th genotype combination. $\alpha_{ij}$are the hyperparameters in a Dirichlet distribution (Peng-Jie Jing, Hong-Bin Shen ,2014; V*isweswaran S,2009*) which define the prior probability over the the parameters $\theta_{M}$, and $\alpha_{i}=\sum_{j=1}^{J} \alpha_{ij}$. We can view the hyperparameters as prior belief numbers of cases from a previous hypothetical study which belongs to the disease state *j* where the genotypes is given by *i-th* combination. When parameters$\alpha_{ij}>0$ *and $n_{ij}>0$, the* Equation S2 (5) can be expressed as follow:

$P(D|M)=\prod_{i=1}^{I} \left(\frac{(\alpha_{i}-1)!}{(n_{i}+\alpha_{i}-1)!}\prod_{j=1}^{J} \frac{(n_{ij}+\alpha_{ij}-1)!}{(\alpha_{ij}-1)!}\right)$ S2(6)

According to the analysis in V*isweswaran S,2009,* for the BN model *M*, it is unknown for us to prior belief of the model M. Thus, we can consider all BN models to be equally plausible and think the prior probability $P\left(M\right)$is a constant for all BN models. Thereby, the Equation S2(3) can be expressed as :

$P(M|D)\propto P(D|M)$ S2(7)

According to Equation S2 (6).

$P(M|D)\propto\prod_{i=1}^{I} \left(\frac{(\alpha_{i}-1)!}{(n_{i}+\alpha_{i}-1)!}\prod_{j=1}^{J} \frac{(n_{ij}+\alpha_{ij}-1)!}{(\alpha_{ij}-1)!}\right)$ S2(8)

Set all $\alpha_{ij}=1$($\alpha_{i}=\sum_{j=1}^{J} \alpha_{ij}=J$), which means that all possible distributions of *Y* given $X=x_{i}$ is equally likely. Thus the Equation S2(8) can be written as

$P(M|D)\propto\prod_{i=1}^{I} \left(\frac{(J-1)!}{(n_{i}+J-1)!}\prod_{j=1}^{J} n_{ij}!\right)$ S2(9)

The [evaluation](javascript:void(0);) [criterion](javascript:void(0);) of BN model turns to the K2-Score as follow formula

$K2-Score=\prod_{i=1}^{I} \left(\frac{(J-1)!}{(n_{i}+J-1)!}\prod_{j=1}^{J} n_{ij}!\right)$ S2(10)

**2. Gini Index**

For example, in Table A1, there are two SNP combination: (*x*_1_ *x*_2_ *x*_3_ ) and (*x*_1_ *x*_4_ *x*_7_). The computational method of impurity of Gini index is as follow.

Table A1. Data set of two SNP combinations and disease state

| *Sample id* | *c* | *x*_1_ *x*_2_ *x*_3_ | y | *Sample id* | *c* | *x*_1_ *x*_4_ *x*_7_ | y |
| --- | --- | --- | --- | --- | --- | --- | --- |
| 1 | *c_1_* | *001* | 1 | 1 | *c_1_* | 011 | 0 |
| 2 | c_2_ | 101 | 0 | 2 | c_3_ | 100 | 1 |
| 3 | c_3_ | 110 | 1 | 3 | c_2_ | 102 | 1 |
| 4 | c_3_ | 110 | 1 | 4 | c_3_ | 100 | 0 |
| 5 | c_2_ | 101 | 1 | 5 | c_2_ | 102 | 0 |
| 6 | c_3_ | 110 | 1 | 6 | c_2_ | 102 | 1 |
| 7 | c_4_ | 201 | 0 | 7 | c_3_ | 100 | 0 |
| 8 | c_4_ | 201 | 0 | 8 | c_3_ | 100 | 1 |

$$P_{1}=\frac{1}{8}=0.125,P_{2}=\frac{2}{8}=0.25,P_{3}=\frac{3}{8}=0.375,P_{4}=\frac{2}{8}=0.25$$

$$\begin{matrix} p_{1,0}=\frac{0}{1}=0,\text{ }p_{1,1}=\frac{1}{1}=1 \\ p_{2,0}=\frac{1}{2}=0.5,\text{ }p_{2,1}=\frac{1}{2}=0.5 \\ p_{3,0}=\frac{0}{3}=0,\text{ }p_{3,1}=\frac{3}{3}=1 \\ p_{4,0}=\frac{2}{2}=1,\text{ }p_{1,1}=\frac{0}{2}=0 \end{matrix}$$

$$\begin{matrix} \Phi\left(L(x_{1}x_{2}x_{3})\right)=0.125\times\left(1-1\right) \\ \text{ }+0.25\times\left(1-\left(0.25+0.25\right)\right) \\ \text{ }+0.375\times\left(1-1\right) \\ \text{ }+0.25\times\left(1-1\right) \\ \text{ }=0.125 \end{matrix}$$

$$\begin{matrix} \Phi\left(L(x_{1}x_{4}x_{7})\right)=0.125\times\left(1-1\right) \\ \text{ }+0.375\times\left(1-\left(\frac{1}{9}+\frac{4}{9}\right)\right) \\ \text{ }+0.5\times\left(1-\left(\frac{9}{16}+\frac{1}{16}\right)\right)\text{ } \\ \text{ }=0.354167 \end{matrix}$$

1. Aflakparast, M., Masoudi-Nejad, A., Bozorgmehr, J. H., & Visweswaran, S. (2014). Informative Bayesian Model Selection: a method for identifying interactions in genome-wide data. Molecular BioSystems, 10(10), 2654-2662.
2. Chen X-W, Anantha G, Lin X（2008）: Improving Bayesian Network Structure Learning with Mutual Information-Based Node Ordering in the K2 Algorithm. IEEE Trans on Knowl and Data Eng 2008, 20:628-640.
3. Cooper GF, Herskovits E (1992). A bayesian method for the induction of probabilistic networks from data. Machine Learning 9(4):309-347.
4. Han, B., Chen, X. W., Talebizadeh, Z., & Xu, H. (2012). Genetic studies of complex human diseases: Characterizing SNP-disease associations using Bayesian networks. BMC systems biology, 6(Suppl 3), S14.
5. Heckerman D, Geiger D, Chickering DM （1995）: Learning Bayesian Networks: The Combination of Knowledge and Statistical Data. Mach Learn 1995, 20:197-243.
6. Jiang, X., Barmada, M. M., & Visweswaran, S. (2010). Identifying genetic interactions in genome‐wide data using Bayesian networks. Genetic epidemiology, 34(6), 575-581.
7. Jiang, X., Neapolitan, R. E., Barmada, M. M., & Visweswaran, S. (2011). Learning genetic epistasis using Bayesian network scoring criteria. BMC bioinformatics, 12(1), 89.
8. Visweswaran S, Wong AI, Barmada MM（2009）: A Bayesian method for identifying genetic interactions. Proceedings of the Fall Symposium of the American Medical Informatics Association 2009, 673-677.
9. Visweswaran, S., Wong, A. K. I., & Barmada, M. M. (2009). A Bayesian method for identifying genetic interactions. In AMIA Annual Symposium Proceedings (Vol. 2009, p. 673). American Medical Informatics Association.
10. Zhang, Y., & Liu, J. S. (2007). Bayesian inference of epistatic interactions in case-control studies （BEAM）. Nature genetics, 39(9), 1167-1173.
