# Supplementary material for: FHSA-SED: Two-Locus Model Detection for Genome-Wide Association Study with Harmony Search Algorithm
Source: PLoS One. 2016 Mar 25;11(3):e0150669. doi: 10.1371/journal.pone.0150669 (PMC4807955; doi:10.1371/journal.pone.0150669)
Supplement: S2 File — (DOC) [file pone.0150669.s002.doc]

**Harmony Search Algorithm**

Harmony search (HS) algorithm is a swarm intelligent optimization algorithm (Z.W. Geem, J. Kim, J. Kim, G. Loganathan, 2001; Z.W. Geem, 2008)[1][2]. It mimics the process of improvising a musical harmony when a music orchestra is aiming at composing the most harmonious melody. For an optimization task, each harmony corresponds to a vector which consists of *k* decision variables. The harmony memory (HM) which consists of HMS harmonies is similar to the population in genetic algorithm (GA), where HMS is called the size of HM. In genome-wide association study (GWAS), we can treat a *k-*way interaction model as a harmony and our goal is to find a best harmony which has strongest association with phenotype. The optimization problem for *k*-way interaction model can be expressed as

where, $x_{i}\in\left\{1,2,\cdots,N\right\}$, *N* is the number of SNPs. if $i\neq j$, then$x_{i}\neq x_{j}$.

The steps of harmony search algorithm are as follows:

**Step 1**. Parameter initialization.

The control parameters of harmony search algorithm are specified, which include HMS, harmony memory considering rate (HMCR), pitch-adjusting rate (PAR), fret width (*fw*) (fret width is called formerly bandwidth: *bw*) and the termination criterion (i.e., the maximum function evaluation times(MaxFEs) ).

**Step 2**. Initializing the harmony memory (HM) and calculating the fitness value of each harmony.

| **For** i=1: HMS $X^{i}=\emptyset$ ;  **For** j=1: ***k***  **While** $a\in X^{i}$ $a=\left\lceil rand(0,1)\times N\right\rceil$ ;  **End**  $X^{i}\leftarrow a$;  **End**  // calculating the fitness value $Score(i)=f(X^{i})$ **;**  **End** |
| --- |

where, rand(0,1) represents a uniformly distributed random number between 0 and 1.

**The harmony memory (HM) consists of HMS harmonies, as follow,**

$$\mathbf{HM}=\left[\begin{matrix} X^{1} \\ X^{2} \\ \vdots\\ X^{\text{HMS}} \end{matrix}\right]=\left[\begin{matrix} x_{1}^{1} & x_{2}^{1} & \cdots& x_{k}^{1} \\ x_{1}^{2} & x_{2}^{2} & \cdots& x_{k}^{2} \\ \vdots& \vdots& \vdots& \vdots\\ x_{1}^{\text{HMS}} & x_{2}^{\text{HMS}} & \cdots& x_{k}^{\text{HMS}} \end{matrix}\left|\begin{matrix} Score(1) \\ Score(2) \\ \vdots\\ Score(\text{HMS}) \end{matrix}\right.\right]$$

**Step 3**. Improvising a new harmony$X^{new}$.

| $X^{new}=\emptyset$ **For** j=1:***k***  **If** *rand (0,1)*<HMCR $X^{new}(j)=\text{HM}\left(\left\lceil rand(0,1)\times\text{HMS}\right\rceil,j\right)$   **If** *rand(0,1)*<PAR $X^{new}(j)=\left\lceil X^{new}(j)\pm fw\right\rceil$   **End**  **Else** $X^{new}(j)=\left\lceil rand(0,1)\times N\right\rceil$ ;  **End**  **End** |
| --- |

**Step 4**. Update operation: updating the worst harmony in HM.

| **If** $f(X^{new})>f(X^{idworst})$ $\begin{matrix} X^{idworst}=X^{new}; \\ Score(idworst)=f(X^{new}); \end{matrix}$   **End** |
| --- |

where $idworst$ is the index of the worst harmony in HM.

**Step 5**. Checking the stopping criterion. If stopping criterion (MaxFEs) is meet, computation is terminated. Otherwise, Step 3 and Step 4 are repeated.

**Fig B1.** The flow chart of harmony search algorithm for the detection of SNP interaction model

1. **Z.W. Geem**, J. Kim, G. Loganathan, [Music-inspired optimization algorithm harmony search](http://sim.sagepub.com/cgi/content/abstract/76/2/60), Simulation, 2001, 76:60-68.
2. **Z.W. Geem**, [Novel Derivative of Harmony Search Algorithm for Discrete Design Variables](http://dx.doi.org/10.1016/j.amc.2007.09.049), Applied Mathematics and Computation, 2008.
