# Supplementary material for: FHSA-SED: Two-Locus Model Detection for Genome-Wide Association Study with Harmony Search Algorithm
Source: PLoS One. 2016 Mar 25;11(3):e0150669. doi: 10.1371/journal.pone.0150669 (PMC4807955; doi:10.1371/journal.pone.0150669)
Supplement: S3 File — All the supplementary experiment data, experiment results and figures. (DOC) [file pone.0150669.s003.doc]

**Experimental data and results**

Table E-1. the parameters and the values of penetrance of 12 DME models

| **DME** | **H^2^** | **MAF** | **D** | **AABB** | **AABb** | **AAbb** | **AaBB** | **AaBb** | **Aabb** | **aaBB** | **aaBb** | **aabb** |
| --- | --- | --- | --- | --- | --- | --- | --- | --- | --- | --- | --- | --- |
| DME -1 | 0.005 | 0.05 | 0.1 | 0.098 | 0.098 | 0.098 | 0.098 | 0.299 | 0.522 | 0.098 | 0.522 | 0.912 |
| DME -2 | 0.005 | 0.1 | 0.1 | 0.096 | 0.096 | 0.096 | 0.096 | 0.197 | 0.282 | 0.096 | 0.282 | 0.405 |
| DME -3 | 0.005 | 0.2 | 0.1 | 0.092 | 0.092 | 0.092 | 0.092 | 0.144 | 0.181 | 0.092 | 0.181 | 0.227 |
| DME -4 | 0.005 | 0.5 | 0.1 | 0.078 | 0.078 | 0.078 | 0.078 | 0.105 | 0.122 | 0.078 | 0.122 | 0.142 |
| DME -5 | 0.02 | 0.05 | 0.1 | 0.096 | 0.096 | 0.096 | 0.096 | 0.533 | 0.533 | 0.096 | 0.533 | 0.533 |
| DME -6 | 0.02 | 0.1 | 0.1 | 0.092 | 0.092 | 0.092 | 0.092 | 0.319 | 0.319 | 0.092 | 0.319 | 0.319 |
| DME -7 | 0.02 | 0.2 | 0.1 | 0.084 | 0.084 | 0.084 | 0.084 | 0.21 | 0.21 | 0.084 | 0.21 | 0.21 |
| DME -8 | 0.02 | 0.5 | 0.1 | 0.052 | 0.052 | 0.052 | 0.052 | 0.137 | 0.137 | 0.052 | 0.137 | 0.137 |
| DME -9 | 0.02 | 0.05 | 0.1 | 0.08 | 0.192 | 0.192 | 0.192 | 0.08 | 0.08 | 0.192 | 0.08 | 0.08 |
| DME -10 | 0.02 | 0.1 | 0.1 | 0.072 | 0.164 | 0.164 | 0.164 | 0.072 | 0.072 | 0.164 | 0.072 | 0.072 |
| DME -11 | 0.02 | 0.2 | 0.1 | 0.061 | 0.146 | 0.146 | 0.146 | 0.061 | 0.061 | 0.146 | 0.061 | 0.061 |
| DME -12 | 0.02 | 0.5 | 0.1 | 0.067 | 0.155 | 0.155 | 0.155 | 0.067 | 0.067 | 0.155 | 0.067 | 0.067 |

Table E-2. the parameters and the values of penetrance of 70 DNME models

| **Model** | **MAF** | **H^2^** | **AABB** | **AABb** | **AAbb** | **AaBB** | **AaBb** | **Aabb** | **aaBB** | **aaBb** | **aabb** |
| --- | --- | --- | --- | --- | --- | --- | --- | --- | --- | --- | --- |
| DNME-1 | 0.20 | 0.40 | 0.4865 | 0.9601 | 0.5377 | 0.9473 | 0.0042 | 0.8113 | 0.6401 | 0.6065 | 0.9089 |
| DNME-2 | 0.20 | 0.40 | 0.4691 | 0.9556 | 0.6973 | 0.9445 | 0.0188 | 0.5848 | 0.7861 | 0.4073 | 0.0127 |
| DNME-3 | 0.20 | 0.40 | 0.4981 | 0.9535 | 0.7864 | 0.9781 | 0.0383 | 0.4276 | 0.5898 | 0.8208 | 0.3801 |
| DNME-4 | 0.20 | 0.40 | 0.5046 | 0.9877 | 0.6242 | 0.9446 | 0.0851 | 0.8068 | 0.9695 | 0.1162 | 0.1594 |
| DNME-5 | 0.20 | 0.40 | 0.4863 | 0.9630 | 0.5119 | 0.9406 | 0.0060 | 0.8990 | 0.6910 | 0.5407 | 0.6143 |
| DNME-6 | 0.20 | 0.20 | 0.4278 | 0.7569 | 0.8117 | 0.7884 | 0.1316 | 0.0439 | 0.5594 | 0.5485 | 0.3730 |
| DNME-7 | 0.20 | 0.20 | 0.5065 | 0.8416 | 0.6054 | 0.8447 | 0.1623 | 0.6285 | 0.5807 | 0.6779 | 0.7289 |
| DNME-8 | 0.20 | 0.20 | 0.5771 | 0.2467 | 0.4282 | 0.2269 | 0.9283 | 0.5783 | 0.5864 | 0.2618 | 0.1582 |
| DNME-9 | 0.20 | 0.20 | 0.3404 | 0.6371 | 0.6539 | 0.6887 | 0.0172 | 0.0414 | 0.2419 | 0.8656 | 0.4031 |
| DNME-10 | 0.20 | 0.20 | 0.3874 | 0.7264 | 0.7340 | 0.7493 | 0.0900 | 0.0344 | 0.5505 | 0.4014 | 0.7238 |
| DNME-11 | 0.20 | 0.10 | 0.4634 | 0.7032 | 0.4311 | 0.6534 | 0.2765 | 0.8056 | 0.8299 | 0.0081 | 0.1293 |
| DNME-12 | 0.20 | 0.10 | 0.3194 | 0.5073 | 0.5686 | 0.5531 | 0.1054 | 0.0448 | 0.2025 | 0.7770 | 0.2804 |
| DNME-13 | 0.20 | 0.10 | 0.6274 | 0.3935 | 0.3353 | 0.3962 | 0.7787 | 0.9533 | 0.3137 | 0.9966 | 0.5301 |
| DNME-14 | 0.20 | 0.10 | 0.2967 | 0.5401 | 0.4412 | 0.5410 | 0.0718 | 0.2776 | 0.4336 | 0.2929 | 0.2282 |
| DNME-15 | 0.20 | 0.10 | 0.3322 | 0.5617 | 0.5731 | 0.5835 | 0.1124 | 0.1474 | 0.3987 | 0.4963 | 0.0332 |
| DNME-16 | 0.20 | 0.05 | 0.4916 | 0.6637 | 0.4807 | 0.6419 | 0.3302 | 0.7457 | 0.6555 | 0.3961 | 0.0000 |
| DNME-17 | 0.20 | 0.05 | 0.4988 | 0.6388 | 0.7649 | 0.6665 | 0.3887 | 0.0831 | 0.5432 | 0.5265 | 0.9533 |
| DNME-18 | 0.20 | 0.05 | 0.2121 | 0.3503 | 0.1161 | 0.3364 | 0.0543 | 0.4948 | 0.2272 | 0.2727 | 0.4948 |
| DNME-19 | 0.20 | 0.05 | 0.8048 | 0.6834 | 0.6378 | 0.6568 | 0.9356 | 0.9890 | 0.8504 | 0.5638 | 0.8656 |
| DNME-20 | 0.20 | 0.05 | 0.6377 | 0.4884 | 0.3826 | 0.4638 | 0.7645 | 0.9566 | 0.5798 | 0.5624 | 0.7189 |
| DNME-21 | 0.20 | 0.30 | 0.4996 | 0.9256 | 0.6149 | 0.8951 | 0.1305 | 0.6469 | 0.8583 | 0.1601 | 0.9992 |
| DNME-22 | 0.20 | 0.30 | 0.4128 | 0.8507 | 0.5355 | 0.8311 | 0.0084 | 0.5801 | 0.6917 | 0.2677 | 0.7363 |
| DNME-23 | 0.20 | 0.30 | 0.4546 | 0.8480 | 0.8973 | 0.8898 | 0.0877 | 0.0165 | 0.5623 | 0.6864 | 0.4666 |
| DNME-24 | 0.20 | 0.30 | 0.6088 | 0.9800 | 0.9800 | 0.9930 | 0.2998 | 0.2747 | 0.8761 | 0.4826 | 0.6830 |
| DNME-25 | 0.20 | 0.30 | 0.4456 | 0.8442 | 0.7739 | 0.8794 | 0.0442 | 0.2330 | 0.4925 | 0.7959 | 0.4104 |
| DNME-26 | 0.40 | 0.40 | 0.0774 | 0.6563 | 0.8804 | 0.8917 | 0.2350 | 0.3120 | 0.1742 | 0.8417 | 0.1064 |
| DNME-27 | 0.40 | 0.40 | 0.8946 | 0.3225 | 0.1608 | 0.0679 | 0.7276 | 0.8058 | 0.9248 | 0.2329 | 0.3619 |
| DNME-28 | 0.40 | 0.40 | 0.8050 | 0.2507 | 0.0847 | 0.0021 | 0.6683 | 0.6383 | 0.8304 | 0.0791 | 0.5423 |
| DNME-29 | 0.40 | 0.40 | 0.3075 | 0.6820 | 0.9579 | 0.9973 | 0.3903 | 0.2809 | 0.0118 | 0.9904 | 0.6977 |
| DNME-30 | 0.40 | 0.40 | 0.0834 | 0.8908 | 0.0371 | 0.6189 | 0.2714 | 0.6907 | 0.8530 | 0.0788 | 0.7417 |
| DNME-31 | 0.40 | 0.20 | 0.3563 | 0.8907 | 0.8085 | 0.9547 | 0.5078 | 0.6110 | 0.6167 | 0.7549 | 0.6304 |
| DNME-32 | 0.40 | 0.20 | 0.0860 | 0.5364 | 0.6413 | 0.6772 | 0.2747 | 0.0965 | 0.2190 | 0.4132 | 0.7117 |
| DNME-33 | 0.40 | 0.20 | 0.8555 | 0.3386 | 0.7723 | 0.5129 | 0.6507 | 0.6069 | 0.2495 | 0.9990 | 0.1545 |
| DNME-34 | 0.40 | 0.20 | 0.5057 | 0.8380 | 0.0235 | 0.6028 | 0.4542 | 0.9566 | 0.7292 | 0.4273 | 0.7527 |
| DNME-35 | 0.40 | 0.20 | 0.3933 | 0.7640 | 0.6636 | 0.8500 | 0.3982 | 0.7333 | 0.4055 | 0.9268 | 0.1475 |
| DNME-36 | 0.40 | 0.10 | 0.1370 | 0.4838 | 0.1869 | 0.4817 | 0.1658 | 0.3654 | 0.1931 | 0.3608 | 0.4298 |
| DNME-37 | 0.40 | 0.10 | 0.4694 | 0.1982 | 0.7542 | 0.3367 | 0.5021 | 0.1411 | 0.3386 | 0.4528 | 0.2847 |
| DNME-38 | 0.40 | 0.10 | 0.4777 | 0.3108 | 0.8635 | 0.3874 | 0.5786 | 0.2633 | 0.6339 | 0.4356 | 0.1378 |
| DNME-39 | 0.40 | 0.10 | 0.0684 | 0.2991 | 0.0171 | 0.2889 | 0.0444 | 0.2852 | 0.0479 | 0.2621 | 0.1743 |
| DNME-40 | 0.40 | 0.10 | 0.5393 | 0.1202 | 0.2579 | 0.1651 | 0.3784 | 0.3254 | 0.1231 | 0.4265 | 0.2755 |
| DNME-41 | 0.40 | 0.05 | 0.0022 | 0.1546 | 0.2141 | 0.1990 | 0.0711 | 0.0219 | 0.0808 | 0.1218 | 0.1355 |
| DNME-42 | 0.40 | 0.05 | 0.1882 | 0.0195 | 0.1714 | 0.0321 | 0.1742 | 0.0585 | 0.1338 | 0.0868 | 0.0920 |
| DNME-43 | 0.40 | 0.05 | 0.0051 | 0.1793 | 0.2510 | 0.2109 | 0.0998 | 0.0265 | 0.1563 | 0.0976 | 0.1563 |
| DNME-44 | 0.40 | 0.05 | 0.1735 | 0.3205 | 0.1542 | 0.2225 | 0.2535 | 0.2450 | 0.4483 | 0.0245 | 0.4242 |
| DNME-45 | 0.40 | 0.05 | 0.0981 | 0.2189 | 0.3020 | 0.3020 | 0.1262 | 0.1214 | 0.0528 | 0.3083 | 0.1359 |
| DNME-46 | 0.40 | 0.30 | 0.8907 | 0.3616 | 0.4796 | 0.2132 | 0.8292 | 0.6014 | 0.9250 | 0.2674 | 0.6852 |
| DNME-47 | 0.40 | 0.30 | 0.0765 | 0.6892 | 0.4165 | 0.7629 | 0.1496 | 0.4909 | 0.1955 | 0.6567 | 0.2465 |
| DNME-48 | 0.40 | 0.30 | 0.1322 | 0.7930 | 0.2738 | 0.7993 | 0.2127 | 0.5137 | 0.2549 | 0.5279 | 0.7930 |
| DNME-49 | 0.40 | 0.30 | 0.6105 | 0.1044 | 0.7592 | 0.1800 | 0.6740 | 0.0189 | 0.5322 | 0.1892 | 0.6810 |
| DNME-50 | 0.40 | 0.30 | 0.0909 | 0.8271 | 0.8631 | 0.8688 | 0.3932 | 0.4145 | 0.7382 | 0.5082 | 0.3634 |
| DNME-51 | 0.20 | 0.03 | 0.4952 | 0.4155 | 0.6571 | 0.4286 | 0.6158 | 0.1214 | 0.5524 | 0.3309 | 0.4190 |
| DNME-52 | 0.20 | 0.03 | 0.5921 | 0.6914 | 0.7433 | 0.7118 | 0.4925 | 0.4189 | 0.5795 | 0.7465 | 0.5040 |
| DNME-53 | 0.20 | 0.03 | 0.1082 | 0.1939 | 0.1860 | 0.1957 | 0.0366 | 0.0454 | 0.1721 | 0.0732 | 0.1304 |
| DNME-54 | 0.20 | 0.03 | 0.1118 | 0.1864 | 0.1281 | 0.1925 | 0.0236 | 0.1383 | 0.0790 | 0.2365 | 0.2508 |
| DNME-55 | 0.20 | 0.03 | 0.2723 | 0.1921 | 0.1848 | 0.1720 | 0.3671 | 0.3896 | 0.3453 | 0.0687 | 0.0049 |
| DNME-56 | 0.20 | 0.01 | 0.2470 | 0.3015 | 0.2049 | 0.3002 | 0.1735 | 0.3777 | 0.2154 | 0.3567 | 0.2680 |
| DNME-57 | 0.20 | 0.01 | 0.2216 | 0.2758 | 0.1414 | 0.2587 | 0.1690 | 0.4013 | 0.2781 | 0.1279 | 0.4196 |
| DNME-58 | 0.20 | 0.01 | 0.2600 | 0.2214 | 0.2011 | 0.2042 | 0.3146 | 0.3483 | 0.3385 | 0.0736 | 0.1276 |
| DNME-59 | 0.20 | 0.01 | 0.1391 | 0.1882 | 0.2214 | 0.1901 | 0.1114 | 0.0198 | 0.2056 | 0.0514 | 0.2530 |
| DNME-60 | 0.20 | 0.01 | 0.5578 | 0.6159 | 0.6740 | 0.6319 | 0.4997 | 0.4184 | 0.5462 | 0.6740 | 0.3951 |
| DNME-61 | 0.40 | 0.03 | 0.1660 | 0.1655 | 0.1277 | 0.1144 | 0.1991 | 0.1431 | 0.2810 | 0.0282 | 0.2810 |
| DNME-62 | 0.40 | 0.03 | 0.1084 | 0.0061 | 0.0799 | 0.0258 | 0.0794 | 0.0457 | 0.0208 | 0.0900 | 0.0252 |
| DNME-63 | 0.40 | 0.03 | 0.0058 | 0.0938 | 0.0077 | 0.0790 | 0.0162 | 0.0759 | 0.0522 | 0.0425 | 0.0570 |
| DNME-64 | 0.40 | 0.03 | 0.1995 | 0.0716 | 0.1675 | 0.0858 | 0.1874 | 0.0760 | 0.1250 | 0.1079 | 0.2260 |
| DNME-65 | 0.40 | 0.03 | 0.1648 | 0.0956 | 0.2619 | 0.1663 | 0.1513 | 0.0915 | 0.0500 | 0.2504 | 0.0559 |
| DNME-66 | 0.40 | 0.01 | 0.1032 | 0.0634 | 0.1242 | 0.0978 | 0.0858 | 0.0693 | 0.0210 | 0.1467 | 0.0595 |
| DNME-67 | 0.40 | 0.01 | 0.1852 | 0.2908 | 0.2340 | 0.2860 | 0.2009 | 0.2770 | 0.2486 | 0.2661 | 0.1657 |
| DNME-68 | 0.40 | 0.01 | 0.0731 | 0.0418 | 0.0146 | 0.0240 | 0.0639 | 0.0591 | 0.0682 | 0.0188 | 0.0946 |
| DNME-69 | 0.40 | 0.01 | 0.0462 | 0.1275 | 0.0694 | 0.1150 | 0.0667 | 0.0971 | 0.1067 | 0.0691 | 0.1085 |
| DNME-70 | 0.40 | 0.01 | 0.0950 | 0.1222 | 0.1267 | 0.0973 | 0.1294 | 0.0999 | 0.2014 | 0.0439 | 0.1222 |

a b

c d

**Fig.E1**. The fitness landscape of DME 1(H^2^=0.005, MAF=0. 05). (a) Fitness curve surface based on K2-scoring criterion. (b) Fitness contour based on K2-scoring criterion. (c) Fitness curve surface based on GINI-scoring criterion. (b) Fitness contour based on GINI-scoring criterion. In the left curve figure, the lower the curve surface, the stronger association with phenotype the genotype combination (SNP1, SNP2) has; in the right contour figure, the deeper the color, the stronger association with phenotype the genotype combination (SNP1, SNP2) has.

a b

c d

Fig.E2. the fitness landscape of DME 8(H^2^=0.02, MAF=0. 5). (a) fitness curve surface based on K2-scoring criterion. (b) Fitness contour based on K2-scoring criterion. (c) Fitness curve surface based on GINI-scoring criterion. (b) Fitness contour based on GINI-scoring criterion. In the right contour figure, the deeper the color, the stronger association with phenotype the genotype combination of (SNP1, SNP2) has. In the left curve figure, the lower the curve surface, the stronger association with phenotype the genotype combination (SNP1, SNP2) has; in the right contour figure, the deeper the color, the stronger association with phenotype the genotype combination (SNP1, SNP2) has.

a b

c d

Fig.E3. The fitness landscape of DME 12(H^2^=0.02, MAF=0. 5). (a) Fitness curve surface based on K2-scoring criterion. (b) Fitness contour based on K2-scoring criterion. (c) Fitness curve surface based on GINI-scoring criterion. (b) Fitness contour based on GINI-scoring criterion. In the left curve figure, the lower the curve surface, the stronger association with phenotype the genotype combination (SNP1, SNP2) has; in the right contour figure, the deeper the color, the stronger association with phenotype the genotype combination (SNP1, SNP2) has.

a  b

c  d

Fig.E4. the fitness landscape of **DNME** (H^2^=0.2, MAF=0. 4). (a) Fitness curve surface based on K2-scoring criterion. (b) Fitness contour based on K2-scoring criterion. (c) Fitness curve surface based on GINI-scoring criterion. (b) Fitness contour based on GINI-scoring criterion. In the left curve figure, the lower the curve surface, the stronger association with phenotype the genotype combination (SNP1, SNP2) has; in the right contour figure, the deeper the color, the stronger association with phenotype the genotype combination (SNP1, SNP2) has.

Fig.E5 The Power comparisons on 70 DNME models: (MAF=0.4; H2=0.01, 0.025, 0.05, 0.1, 0.2, 0.3, 0.4).

Fig.E6 The Power comparisons on 70 DNME models: (MAF=0.4; H2=0.01, 0.025, 0.05, 0.1, 0.2, 0.3, 0.4) .

**Table E-3** The Powers mean Evaluation times and mean computational time on 70 DNME models (100 SNP markers)

|  | **Power** | | | | | | | **Evaluation Times** | | | **Mean time** | |  |
| --- | --- | --- | --- | --- | --- | --- | --- | --- | --- | --- | --- | --- | --- |
| **Model** | **HS+** | **HS+** | **1^st^** | **FHSA-SED** | **1^st^** | **MACOED** | **CSE** | **FHSA-SED** | **MACOED** | **CSE** | **FHSA-SED** | **MACOED** | **CSE** |
|  | **(K2-Score)** | **(Gini-Score)** | **FHSA-SED** |  | **MACOED** |  |  |  |  |  |  |  |  |
| **DNME-1** | 1.0 | 1.0 | 1.0 | 1.0 | 0.9 | 0.9 | 0.3 | 739 | 2459 | 4429 | 1.4 | 31.9 | 22.3 |
| **DNME-2** | 1.0 | 1.0 | 1.0 | 0.2 | 0.5 | 0.1 | 0.3 | 799 | 2526 | 4353 | 1.1 | 32.2 | 24.2 |
| **DNME-3** | 1.0 | 1.0 | 1.0 | 1.0 | 0.9 | 0.9 | 0.2 | 1092 | 2601 | 4642 | 1.6 | 32.8 | 24.2 |
| **DNME-4** | 1.0 | 1.0 | 1.0 | 0.9 | 0.9 | 0.8 | 0.1 | 2120 | 2510 | 4338 | 2.7 | 31.7 | 22.4 |
| **DNME-5** | 0.9 | 0.9 | 0.9 | 0.9 | 0.9 | 0.7 | 0.2 | 2227 | 2517 | 4696 | 2.8 | 31.8 | 25.9 |
| **DNME-6** | 0.9 | 0.9 | 0.9 | 0.9 | 1.0 | 1.0 | 0.2 | 2227 | 2177 | 4546 | 2.8 | 27.4 | 23.1 |
| **DNME-7** | 1.0 | 1.0 | 1.0 | 1.0 | 1.0 | 1.0 | 0.2 | 2205 | 2525 | 4462 | 2.8 | 31.8 | 21.0 |
| **DNME-8** | 0.9 | 0.9 | 0.9 | 0.9 | 0.9 | 0.9 | 0.3 | 2516 | 2496 | 4342 | 3.2 | 31.6 | 22.6 |
| **DNME-9** | 1.0 | 1.0 | 1.0 | 1.0 | 0.9 | 0.9 | 0.1 | 2305 | 2584 | 4266 | 2.9 | 32.9 | 21.6 |
| **DNME-10** | 1.0 | 1.0 | 1.0 | 1.0 | 0.9 | 0.9 | 0.2 | 2256 | 2545 | 4472 | 3.0 | 33.2 | 23.8 |
| **DNME-11** | 0.9 | 0.9 | 0.9 | 0.9 | 0.9 | 0.9 | 0.2 | 2392 | 2449 | 4622 | 3.2 | 30.6 | 23.7 |
| **DNME-12** | 0.9 | 0.9 | 0.9 | 0.9 | 0.9 | 0.9 | 0.2 | 2109 | 2214 | 4582 | 2.6 | 27.9 | 21.4 |
| **DNME-13** | 0.9 | 0.9 | 0.9 | 0.9 | 0.9 | 0.9 | 0.2 | 2238 | 2481 | 4526 | 2.8 | 31.4 | 23.7 |
| **DNME-14** | 0.9 | 0.9 | 0.9 | 0.9 | 0.9 | 0.9 | 0.1 | 2114 | 2715 | 4139 | 2.9 | 34.0 | 21.5 |
| **DNME-15** | 1.0 | 1.0 | 1.0 | 1.0 | 0.9 | 0.9 | 0.2 | 2392 | 2555 | 4668 | 3.2 | 31.9 | 24.8 |
| **DNME-16** | 1.0 | 1.0 | 1.0 | 1.0 | 0.9 | 0.9 | 0.2 | 2414 | 2330 | 4371 | 3.3 | 29.8 | 22.4 |
| **DNME-17** | 1.0 | 1.0 | 1.0 | 1.0 | 0.9 | 0.9 | 0.2 | 2143 | 2488 | 4529 | 3.0 | 31.5 | 24.1 |
| **DNME-18** | 1.0 | 1.0 | 1.0 | 1.0 | 0.9 | 0.9 | 0.2 | 2187 | 2477 | 4764 | 2.8 | 31.6 | 25.8 |
| **DNME-19** | 1.0 | 1.0 | 1.0 | 1.0 | 0.9 | 0.9 | 0.1 | 2322 | 2388 | 4310 | 3.0 | 30.3 | 22.5 |
| **DNME-20** | 1.0 | 1.0 | 1.0 | 1.0 | 0.9 | 0.9 | 0.1 | 2234 | 2613 | 4716 | 2.9 | 32.6 | 25.1 |
| **DNME-21** | 1.0 | 1.0 | 1.0 | 1.0 | 1.0 | 1.0 | 0.2 | 1966 | 2369 | 4540 | 3.1 | 30.8 | 23.0 |
| **DNME-22** | 1.0 | 1.0 | 1.0 | 1.0 | 0.9 | 0.9 | 0.2 | 2167 | 2390 | 4514 | 3.3 | 30.7 | 24.1 |
| **DNME-23** | 1.0 | 1.0 | 1.0 | 1.0 | 0.9 | 0.9 | 0.2 | 2259 | 2572 | 4694 | 3.3 | 32.9 | 25.5 |
| **DNME-24** | 1.0 | 1.0 | 1.0 | 1.0 | 0.9 | 0.9 | 0.1 | 2246 | 2464 | 4350 | 3.3 | 31.6 | 23.0 |
| **DNME-25** | 0.9 | 0.9 | 0.9 | 0.9 | 1.0 | 1.0 | 0.3 | 2229 | 2350 | 4459 | 3.1 | 30.9 | 23.8 |
| **DNME-26** | 0.9 | 0.9 | 0.9 | 0.9 | 0.9 | 0.9 | 0.2 | 2407 | 2669 | 4460 | 3.3 | 34.9 | 22.6 |
| **DNME-27** | 1.0 | 1.0 | 1.0 | 1.0 | 0.9 | 0.9 | 0.2 | 2350 | 2672 | 4541 | 3.4 | 34.8 | 24.8 |
| **DNME-28** | 0.9 | 0.9 | 0.9 | 0.9 | 0.9 | 0.9 | 0.2 | 2366 | 2603 | 4525 | 3.3 | 33.3 | 24.1 |
| **DNME-29** | 0.9 | 0.9 | 0.9 | 0.9 | 0.9 | 0.9 | 0.2 | 2354 | 2690 | 4152 | 3.3 | 34.2 | 21.6 |
| **DNME-30** | 0.9 | 0.9 | 0.9 | 0.9 | 0.9 | 0.9 | 0.2 | 2360 | 2436 | 4477 | 3.3 | 32.1 | 24.0 |
| **DNME-31** | 1.0 | 1.0 | 1.0 | 1.0 | 0.9 | 0.9 | 0.2 | 2172 | 2878 | 4627 | 3.1 | 37.0 | 23.8 |
| **DNME-32** | 1.0 | 1.0 | 1.0 | 1.0 | 0.9 | 0.9 | 0.2 | 2350 | 2613 | 4634 | 3.2 | 33.0 | 25.8 |
| **DNME-33** | 0.9 | 0.9 | 0.9 | 0.9 | 0.9 | 0.9 | 0.1 | 2462 | 2610 | 4755 | 3.2 | 33.7 | 25.3 |
| **DNME-34** | 0.9 | 0.9 | 0.9 | 0.9 | 1.0 | 1.0 | 0.1 | 2414 | 2325 | 4397 | 3.1 | 29.6 | 22.8 |
| **DNME-35** | 1.0 | 1.0 | 1.0 | 1.0 | 0.9 | 0.9 | 0.2 | 2206 | 2608 | 4594 | 2.7 | 34.3 | 23.4 |
| **DNME-36** | 0.9 | 0.9 | 0.9 | 0.5 | 0.4 | 0.1 | 0.2 | 2506 | 2475 | 4516 | 3.2 | 32.0 | 22.0 |
| **DNME-37** | 0.9 | 0.9 | 0.9 | 0.7 | 0.6 | 0.1 | 0.2 | 2299 | 2590 | 4363 | 2.8 | 32.8 | 23.5 |
| **DNME-38** | 0.9 | 0.9 | 0.9 | 0.6 | 0.5 | 0.1 | 0.2 | 2286 | 2523 | 4570 | 2.8 | 32.9 | 23.9 |
| **DNME-39** | 0.9 | 0.9 | 0.9 | 0.7 | 0.9 | 0.4 | 0.1 | 2237 | 2511 | 4329 | 2.8 | 32.5 | 21.9 |
| **DNME-40** | 0.7 | 0.8 | 0.8 | 0.4 | 0.5 | 0.1 | 0.2 | 2670 | 2396 | 4622 | 3.3 | 31.2 | 21.1 |
| **DNME-41** | 0.9 | 0.9 | 0.9 | 0.9 | 0.9 | 0.9 | 0.2 | 2224 | 2565 | 4556 | 2.7 | 33.0 | 22.5 |
| **DNME-42** | 1.0 | 1.0 | 1.0 | 1.0 | 1.0 | 1.0 | 0.2 | 2149 | 2243 | 4568 | 2.6 | 28.4 | 25.3 |
| **DNME-43** | 0.9 | 0.9 | 0.9 | 0.9 | 0.9 | 0.9 | 0.3 | 2330 | 2428 | 4275 | 2.7 | 31.9 | 22.7 |
| **DNME-44** | 0.9 | 0.9 | 0.9 | 0.9 | 0.9 | 0.9 | 0.1 | 2532 | 2549 | 4244 | 3.0 | 32.6 | 21.4 |
| **DNME-45** | 0.9 | 0.9 | 0.9 | 0.9 | 0.9 | 0.9 | 0.3 | 2298 | 2515 | 4516 | 2.8 | 32.5 | 19.7 |
| **DNME-46** | 1.0 | 1.0 | 1.0 | 0.9 | 0.9 | 0.7 | 0.3 | 2321 | 2355 | 4428 | 3.1 | 30.0 | 21.8 |
| **DNME-47** | 0.9 | 0.9 | 0.9 | 0.9 | 0.9 | 0.6 | 0.2 | 2292 | 2464 | 4566 | 2.8 | 31.9 | 24.0 |
| **DNME-48** | 0.9 | 0.9 | 0.9 | 0.9 | 0.9 | 0.9 | 0.2 | 2383 | 2569 | 4424 | 3.2 | 33.2 | 23.2 |
| **DNME-49** | 0.8 | 0.8 | 0.8 | 0.8 | 0.9 | 0.9 | 0.2 | 2560 | 2660 | 4431 | 3.2 | 32.8 | 23.2 |
| **DNME-50** | 0.9 | 0.9 | 0.9 | 0.9 | 0.9 | 0.9 | 0.2 | 2449 | 2454 | 4534 | 3.1 | 29.8 | 19.8 |
| **DNME-51** | 0.9 | 0.9 | 0.9 | 0.9 | 0.9 | 0.9 | 0.2 | 2297 | 2342 | 4623 | 3.1 | 29.2 | 23.3 |
| **DNME-52** | 0.9 | 0.9 | 0.9 | 0.9 | 0.9 | 0.9 | 0.2 | 2511 | 2567 | 4468 | 3.5 | 33.3 | 23.7 |
| **DNME-53** | 0.9 | 0.9 | 0.9 | 0.9 | 0.9 | 0.9 | 0.3 | 2476 | 2410 | 4479 | 3.1 | 31.3 | 23.0 |
| **DNME-54** | 0.9 | 0.9 | 0.9 | 0.9 | 0.9 | 0.9 | 0.2 | 2460 | 2319 | 4263 | 3.5 | 28.6 | 21.6 |
| **DNME-55** | 0.9 | 0.9 | 0.9 | 0.9 | 0.9 | 0.9 | 0.2 | 2309 | 2539 | 4727 | 3.1 | 30.8 | 21.0 |
| **DNME-56** | 0.9 | 0.9 | 0.9 | 0.9 | 0.9 | 0.9 | 0.2 | 2425 | 2613 | 4651 | 2.9 | 32.0 | 23.6 |
| **DNME-57** | 0.9 | 0.9 | 0.9 | 0.9 | 1.0 | 1.0 | 0.2 | 2177 | 2500 | 4730 | 2.6 | 32.6 | 25.7 |
| **DNME-58** | 0.9 | 0.9 | 0.9 | 0.9 | 0.9 | 0.9 | 0.2 | 2543 | 2585 | 4557 | 3.1 | 34.0 | 22.9 |
| **DNME-59** | 1.0 | 1.0 | 1.0 | 1.0 | 0.9 | 0.9 | 0.2 | 2270 | 2528 | 4319 | 2.7 | 31.1 | 21.4 |
| **DNME-60** | 0.9 | 0.9 | 0.9 | 0.9 | 0.9 | 0.9 | 0.2 | 2006 | 2376 | 4667 | 2.5 | 31.9 | 20.8 |
| **DNME-61** | 0.9 | 0.9 | 0.9 | 0.9 | 0.9 | 0.9 | 0.2 | 2345 | 2399 | 4554 | 3.1 | 29.6 | 22.8 |
| **DNME-62** | 0.9 | 0.9 | 0.9 | 0.9 | 0.9 | 0.9 | 0.3 | 2301 | 2366 | 4305 | 3.0 | 30.5 | 22.5 |
| **DNME-63** | 0.9 | 0.9 | 0.9 | 0.9 | 0.9 | 0.9 | 0.1 | 2310 | 2653 | 4795 | 3.0 | 34.7 | 25.7 |
| **DNME-64** | 0.9 | 0.9 | 0.9 | 0.9 | 0.9 | 0.9 | 0.2 | 2145 | 2548 | 4257 | 2.8 | 31.3 | 22.3 |
| **DNME-65** | 0.9 | 0.9 | 0.9 | 0.9 | 0.9 | 0.9 | 0.3 | 2520 | 2508 | 4393 | 3.2 | 32.8 | 19.3 |
| **DNME-66** | 1.0 | 1.0 | 1.0 | 1.0 | 0.8 | 0.8 | 0.2 | 2041 | 2615 | 4573 | 2.6 | 32.6 | 23.6 |
| **DNME-67** | 0.9 | 0.9 | 0.9 | 0.9 | 0.9 | 0.9 | 0.2 | 2302 | 2423 | 4388 | 3.0 | 31.1 | 22.7 |
| **DNME-68** | 0.9 | 0.9 | 0.9 | 0.9 | 0.9 | 0.9 | 0.2 | 2327 | 2576 | 4712 | 3.1 | 33.5 | 24.2 |
| **DNME-69** | 0.9 | 0.9 | 0.9 | 0.9 | 0.9 | 0.9 | 0.2 | 2499 | 2586 | 4212 | 3.3 | 31.2 | 21.7 |
| **DNME-70** | 0.9 | 0.9 | 0.9 | 0.9 | 0.9 | 0.9 | 0.3 | 2495 | 2522 | 4534 | 3.2 | 32.3 | 19.8 |

Table E-4 Six performance metrics (TPR, SPC, PPV, FDR, ACC, F1-Score) for two algorithms: FHSA-SED and MACOED models (100 SNP markers)

|  | **FHSA-SED** | | | | | | **MACOED** | | | | | |
| --- | --- | --- | --- | --- | --- | --- | --- | --- | --- | --- | --- | --- |
| **Model** | **TPR** | **SPC** | **PPV** | **FDR** | **ACC** | **F1-Score** | **TPR** | **SPC** | **PPV** | **FDR** | **ACC** | **F1-Score** |
| **DNME-1** | 99% | 100% | 100% | 0% | 100% | 99% | 100% | 100% | 100% | 0% | 100% | 100% |
| **DNME-2** | 23% | 100% | 99% | 1% | 99% | 38% | 100% | 100% | 100% | 0% | 100% | 100% |
| **DNME-3** | 100% | 100% | 100% | 0% | 100% | 100% | 100% | 100% | 100% | 0% | 100% | 100% |
| **DNME-4** | 95% | 100% | 100% | 0% | 100% | 97% | 100% | 100% | 100% | 0% | 100% | 100% |
| **DNME-5** | 91% | 100% | 100% | 0% | 100% | 95% | 100% | 100% | 100% | 0% | 100% | 100% |
| **DNME-6** | 100% | 100% | 100% | 0% | 100% | 100% | 100% | 100% | 99% | 1% | 100% | 99% |
| **DNME-7** | 100% | 100% | 100% | 0% | 100% | 100% | 100% | 100% | 100% | 0% | 100% | 100% |
| **DNME-8** | 100% | 99% | 99% | 1% | 99% | 100% | 100% | 100% | 100% | 0% | 100% | 100% |
| **DNME-9** | 100% | 100% | 100% | 0% | 100% | 100% | 100% | 99% | 99% | 1% | 100% | 99% |
| **DNME-10** | 100% | 100% | 100% | 0% | 100% | 100% | 100% | 100% | 99% | 1% | 100% | 99% |
| **DNME-11** | 100% | 100% | 100% | 0% | 100% | 100% | 100% | 100% | 100% | 0% | 100% | 100% |
| **DNME-12** | 100% | 100% | 100% | 0% | 100% | 100% | 100% | 100% | 100% | 0% | 100% | 100% |
| **DNME-13** | 100% | 100% | 100% | 0% | 100% | 100% | 100% | 100% | 100% | 0% | 100% | 100% |
| **DNME-14** | 100% | 100% | 100% | 0% | 100% | 100% | 100% | 100% | 99% | 1% | 100% | 99% |
| **DNME-15** | 100% | 100% | 100% | 0% | 100% | 100% | 100% | 100% | 100% | 0% | 100% | 100% |
| **DNME-16** | 100% | 100% | 100% | 0% | 100% | 100% | 100% | 100% | 100% | 0% | 100% | 100% |
| **DNME-17** | 100% | 100% | 100% | 0% | 100% | 100% | 100% | 100% | 100% | 0% | 100% | 100% |
| **DNME-18** | 100% | 100% | 100% | 0% | 100% | 100% | 100% | 96% | 99% | 1% | 99% | 99% |
| **DNME-19** | 100% | 100% | 100% | 0% | 100% | 100% | 100% | 100% | 100% | 0% | 100% | 100% |
| **DNME-20** | 100% | 100% | 100% | 0% | 100% | 100% | 100% | 100% | 100% | 0% | 100% | 100% |
| **DNME-21** | 100% | 100% | 100% | 0% | 100% | 100% | 100% | 100% | 100% | 0% | 100% | 100% |
| **DNME-22** | 100% | 100% | 100% | 0% | 100% | 100% | 100% | 100% | 100% | 0% | 100% | 100% |
| **DNME-23** | 100% | 100% | 100% | 0% | 100% | 100% | 100% | 100% | 100% | 0% | 100% | 100% |
| **DNME-24** | 100% | 100% | 100% | 0% | 100% | 100% | 100% | 100% | 100% | 0% | 100% | 100% |
| **DNME-25** | 100% | 100% | 100% | 0% | 100% | 100% | 100% | 100% | 100% | 0% | 100% | 100% |
| **DNME-26** | 100% | 100% | 100% | 0% | 100% | 100% | 100% | 100% | 100% | 0% | 100% | 100% |
| **DNME-27** | 100% | 100% | 100% | 0% | 100% | 100% | 100% | 100% | 100% | 0% | 100% | 100% |
| **DNME-28** | 100% | 99% | 99% | 1% | 99% | 100% | 100% | 100% | 99% | 1% | 100% | 99% |
| **DNME-29** | 100% | 100% | 100% | 0% | 100% | 100% | 100% | 100% | 99% | 1% | 100% | 99% |
| **DNME-30** | 100% | 99% | 99% | 1% | 99% | 100% | 100% | 100% | 100% | 0% | 100% | 100% |
| **DNME-31** | 100% | 100% | 100% | 0% | 100% | 100% | 100% | 97% | 99% | 1% | 99% | 99% |
| **DNME-32** | 100% | 100% | 100% | 0% | 100% | 100% | 100% | 100% | 100% | 0% | 100% | 100% |
| **DNME-33** | 100% | 99% | 99% | 1% | 99% | 100% | 100% | 100% | 100% | 0% | 100% | 100% |
| **DNME-34** | 100% | 100% | 100% | 0% | 100% | 100% | 100% | 100% | 100% | 0% | 100% | 100% |
| **DNME-35** | 100% | 100% | 100% | 0% | 100% | 100% | 100% | 100% | 100% | 0% | 100% | 100% |
| **DNME-36** | 56% | 84% | 78% | 22% | 84% | 65% | 100% | 100% | 88% | 13% | 100% | 93% |
| **DNME-37** | 80% | 86% | 85% | 15% | 86% | 82% | 100% | 100% | 100% | 0% | 100% | 100% |
| **DNME-38** | 66% | 85% | 81% | 19% | 85% | 73% | 100% | 100% | 89% | 11% | 100% | 94% |
| **DNME-39** | 81% | 86% | 85% | 15% | 86% | 83% | 100% | 100% | 100% | 0% | 100% | 100% |
| **DNME-40** | 52% | 85% | 78% | 22% | 85% | 62% | 100% | 100% | 100% | 0% | 100% | 100% |
| **DNME-41** | 100% | 87% | 89% | 11% | 87% | 94% | 100% | 100% | 100% | 0% | 100% | 100% |
| **DNME-42** | 100% | 85% | 87% | 13% | 86% | 93% | 100% | 100% | 100% | 0% | 100% | 100% |
| **DNME-43** | 100% | 84% | 86% | 14% | 84% | 93% | 100% | 99% | 99% | 1% | 100% | 99% |
| **DNME-44** | 100% | 85% | 87% | 13% | 85% | 93% | 100% | 100% | 100% | 0% | 100% | 100% |
| **DNME-45** | 100% | 85% | 87% | 13% | 85% | 93% | 100% | 100% | 100% | 0% | 100% | 100% |
| **DNME-46** | 98% | 86% | 88% | 12% | 87% | 93% | 100% | 100% | 100% | 0% | 100% | 100% |
| **DNME-47** | 97% | 86% | 87% | 13% | 86% | 92% | 100% | 99% | 98% | 2% | 99% | 99% |
| **DNME-48** | 100% | 85% | 87% | 13% | 85% | 93% | 100% | 100% | 100% | 0% | 100% | 100% |
| **DNME-49** | 100% | 86% | 88% | 12% | 86% | 93% | 100% | 100% | 100% | 0% | 100% | 100% |
| **DNME-50** | 100% | 86% | 87% | 13% | 86% | 93% | 100% | 99% | 99% | 1% | 100% | 99% |
| **DNME-51** | 100% | 85% | 87% | 13% | 86% | 93% | 100% | 100% | 100% | 0% | 100% | 100% |
| **DNME-52** | 100% | 84% | 87% | 13% | 85% | 93% | 100% | 100% | 100% | 0% | 100% | 100% |
| **DNME-53** | 100% | 85% | 87% | 13% | 85% | 93% | 100% | 100% | 100% | 0% | 100% | 100% |
| **DNME-54** | 100% | 85% | 87% | 13% | 86% | 93% | 100% | 100% | 100% | 0% | 100% | 100% |
| **DNME-55** | 100% | 85% | 87% | 13% | 85% | 93% | 100% | 100% | 100% | 0% | 100% | 100% |
| **DNME-56** | 100% | 85% | 87% | 13% | 85% | 93% | 100% | 100% | 100% | 0% | 100% | 100% |
| **DNME-57** | 100% | 88% | 89% | 11% | 88% | 94% | 100% | 100% | 100% | 0% | 100% | 100% |
| **DNME-58** | 100% | 84% | 86% | 14% | 84% | 93% | 100% | 100% | 100% | 0% | 100% | 100% |
| **DNME-59** | 100% | 86% | 88% | 12% | 86% | 94% | 100% | 100% | 100% | 0% | 100% | 100% |
| **DNME-60** | 100% | 86% | 88% | 12% | 86% | 93% | 100% | 100% | 100% | 0% | 100% | 100% |
| **DNME-61** | 100% | 86% | 87% | 13% | 86% | 93% | 100% | 100% | 100% | 0% | 100% | 100% |
| **DNME-62** | 100% | 85% | 87% | 13% | 85% | 93% | 100% | 100% | 100% | 0% | 100% | 100% |
| **DNME-63** | 100% | 83% | 86% | 14% | 83% | 92% | 100% | 100% | 100% | 0% | 100% | 100% |
| **DNME-64** | 100% | 86% | 88% | 12% | 86% | 93% | 100% | 100% | 100% | 0% | 100% | 100% |
| **DNME-65** | 100% | 84% | 86% | 14% | 84% | 93% | 100% | 100% | 100% | 0% | 100% | 100% |
| **DNME-66** | 100% | 86% | 87% | 13% | 86% | 93% | 100% | 100% | 100% | 0% | 100% | 100% |
| **DNME-67** | 100% | 85% | 87% | 13% | 85% | 93% | 100% | 100% | 100% | 0% | 100% | 100% |
| **DNME-68** | 100% | 84% | 86% | 14% | 84% | 92% | 100% | 100% | 100% | 0% | 100% | 100% |
| **DNME-69** | 100% | 86% | 87% | 13% | 86% | 93% | 100% | 98% | 99% | 1% | 99% | 99% |
| **DNME-70** | 100% | 84% | 86% | 14% | 84% | 93% | 100% | 100% | 100% | 0% | 100% | 100% |

Fig.E7 the Power on 70 DNME models with 1000 SNP markers

Fig.E8 the Computational times and Evaluation times on 70 DME models with 1000 SNP markers for FHSA-SED and Exhaustive search algorithm

**Table E-5 the performance (TPR, SPC, PPV, FDR, ACC, F1-Score), Power, Evaluation times and computational time for FHSA-SED**

| **1000SNP** | **TPR** | **SPC** | **PPV** | **FDR** | **ACC** | **F1** | **Power** | | | | **Evaluation Times** | **Mean time** |
| --- | --- | --- | --- | --- | --- | --- | --- | --- | --- | --- | --- | --- |
|  |  |  |  |  |  |  | **K2** | **GINI** | **1^st^ FHSA-SED** | **FHSA-SED** |  |  |
| DNME-1 | 94.00% | 98.33% | 98.25% | 1.75% | 98.31% | 35.01% | 1.00 | 1.00 | 1.00 | 0.94 | 81970.90 | 213.63 |
| DNME-2 | 10.61% | 98.21% | 85.56% | 14.44% | 97.93% | 3.17% | 0.66 | 0.66 | 0.66 | 0.07 | 160862.96 | 423.42 |
| DNME-3 | 100.00% | 98.60% | 98.62% | 1.38% | 98.61% | 40.98% | 1.00 | 1.00 | 1.00 | 1.00 | 87206.81 | 229.35 |
| DNME-4 | 72.00% | 98.62% | 98.12% | 1.88% | 98.49% | 31.58% | 1.00 | 1.00 | 1.00 | 0.72 | 86762.68 | 226.09 |
| DNME-5 | 57.14% | 98.54% | 97.51% | 2.49% | 98.35% | 24.72% | 0.98 | 0.98 | 0.98 | 0.56 | 91245.92 | 236.17 |
| DNME-6 | 100.00% | 98.48% | 98.51% | 1.49% | 98.49% | 39.14% | 1.00 | 1.00 | 1.00 | 1.00 | 84590.01 | 217.47 |
| DNME-7 | 100.00% | 98.55% | 98.57% | 1.43% | 98.55% | 40.16% | 1.00 | 1.00 | 1.00 | 1.00 | 85417.06 | 227.90 |
| DNME-8 | 100.00% | 98.48% | 98.50% | 1.50% | 98.49% | 39.06% | 1.00 | 1.00 | 1.00 | 1.00 | 88076.26 | 226.98 |
| DNME-9 | 99.00% | 98.67% | 98.67% | 1.33% | 98.67% | 41.95% | 1.00 | 1.00 | 1.00 | 0.99 | 86108.21 | 223.60 |
| DNME-10 | 100.00% | 98.46% | 98.48% | 1.52% | 98.46% | 38.68% | 1.00 | 1.00 | 1.00 | 1.00 | 87531.98 | 224.15 |
| DNME-11 | 100.00% | 98.86% | 98.87% | 1.13% | 98.86% | 45.98% | 1.00 | 1.00 | 1.00 | 1.00 | 86371.20 | 235.05 |
| DNME-12 | 100.00% | 98.47% | 98.50% | 1.50% | 98.48% | 38.99% | 1.00 | 1.00 | 1.00 | 1.00 | 83355.69 | 226.32 |
| DNME-13 | 100.00% | 98.47% | 98.50% | 1.50% | 98.48% | 38.91% | 1.00 | 1.00 | 1.00 | 1.00 | 86103.85 | 222.11 |
| DNME-14 | 100.00% | 96.22% | 96.35% | 3.65% | 96.29% | 51.02% | 1.00 | 1.00 | 1.00 | 1.00 | 76974.05 | 207.31 |
| DNME-15 | 100.00% | 95.83% | 96.00% | 4.00% | 95.91% | 48.54% | 1.00 | 1.00 | 1.00 | 1.00 | 77467.39 | 220.00 |
| DNME-16 | 100.00% | 98.45% | 98.47% | 1.53% | 98.46% | 38.61% | 1.00 | 1.00 | 1.00 | 1.00 | 87032.82 | 213.22 |
| DNME-17 | 100.00% | 95.70% | 95.88% | 4.12% | 95.78% | 47.73% | 1.00 | 1.00 | 1.00 | 1.00 | 75066.82 | 203.08 |
| DNME-18 | 100.00% | 95.33% | 95.53% | 4.47% | 95.42% | 45.66% | 1.00 | 1.00 | 1.00 | 1.00 | 78681.68 | 212.67 |
| DNME-19 | 100.00% | 95.34% | 95.54% | 4.46% | 95.43% | 97.72% | 1.00 | 1.00 | 1.00 | 1.00 | 75908.76 | 206.50 |
| DNME-20 | 100.00% | 95.72% | 95.89% | 4.11% | 95.80% | 97.90% | 1.00 | 1.00 | 1.00 | 1.00 | 77227.52 | 211.47 |
| DNME-21 | 100.00% | 95.73% | 95.91% | 4.09% | 95.82% | 97.91% | 1.00 | 1.00 | 1.00 | 1.00 | 78694.39 | 212.40 |
| DNME-22 | 100.00% | 97.99% | 98.03% | 1.97% | 98.03% | 99.01% | 1.00 | 1.00 | 1.00 | 1.00 | 27522.58 | 35.40 |
| DNME-23 | 100.00% | 98.02% | 98.06% | 1.94% | 98.06% | 99.02% | 1.00 | 1.00 | 1.00 | 1.00 | 26162.65 | 33.72 |
| DNME-24 | 100.00% | 95.39% | 95.60% | 4.40% | 95.48% | 97.75% | 1.00 | 1.00 | 1.00 | 1.00 | 75881.34 | 211.89 |
| DNME-25 | 100.00% | 96.01% | 96.16% | 3.84% | 96.08% | 98.04% | 1.00 | 1.00 | 1.00 | 1.00 | 76664.07 | 215.84 |
| DNME-26 | 100.00% | 95.88% | 96.04% | 3.96% | 95.96% | 97.98% | 1.00 | 1.00 | 1.00 | 1.00 | 76121.95 | 215.87 |
| DNME-27 | 100.00% | 95.63% | 95.81% | 4.19% | 95.71% | 97.86% | 1.00 | 1.00 | 1.00 | 1.00 | 77956.14 | 221.71 |
| DNME-28 | 100.00% | 95.14% | 95.37% | 4.63% | 95.23% | 97.63% | 1.00 | 1.00 | 1.00 | 1.00 | 74245.86 | 207.95 |
| DNME-29 | 100.00% | 95.81% | 95.97% | 4.03% | 95.89% | 97.95% | 1.00 | 1.00 | 1.00 | 1.00 | 75928.95 | 198.09 |
| DNME-30 | 100.00% | 94.57% | 94.85% | 5.15% | 94.68% | 97.36% | 1.00 | 1.00 | 1.00 | 1.00 | 76399.78 | 199.98 |
| DNME-31 | 100.00% | 97.99% | 98.03% | 1.97% | 98.03% | 99.01% | 1.00 | 1.00 | 1.00 | 1.00 | 28596.82 | 37.27 |
| DNME-32 | 100.00% | 97.97% | 98.01% | 1.99% | 98.01% | 99.00% | 1.00 | 1.00 | 1.00 | 1.00 | 30726.45 | 39.64 |
| DNME-33 | 100.00% | 97.95% | 97.99% | 2.01% | 97.99% | 98.99% | 1.00 | 1.00 | 1.00 | 1.00 | 26716.11 | 35.63 |
| DNME-34 | 100.00% | 98.01% | 98.04% | 1.96% | 98.04% | 99.01% | 1.00 | 1.00 | 1.00 | 1.00 | 26720.94 | 33.61 |
| DNME-35 | 100.00% | 97.97% | 98.01% | 1.99% | 98.01% | 99.00% | 1.00 | 1.00 | 1.00 | 1.00 | 27033.07 | 33.51 |
| DNME-36 | 4.84% | 96.40% | 57.37% | 42.63% | 95.39% | 8.92% | 0.61 | 0.61 | 0.62 | 0.03 | 154656.05 | 203.39 |
| DNME-37 | 24.29% | 96.68% | 87.98% | 12.02% | 95.78% | 38.06% | 0.68 | 0.70 | 0.70 | 0.17 | 126877.06 | 161.44 |
| DNME-38 | 12.50% | 96.17% | 76.53% | 23.47% | 95.10% | 21.49% | 0.70 | 0.70 | 0.72 | 0.09 | 131256.75 | 162.71 |
| DNME-39 | 58.33% | 97.07% | 95.22% | 4.78% | 96.40% | 72.35% | 0.96 | 0.95 | 0.96 | 0.56 | 47438.98 | 60.75 |
| DNME-40 | 1.79% | 96.36% | 32.91% | 67.09% | 95.41% | 3.39% | 0.56 | 0.55 | 0.56 | 0.01 | 167764.68 | 222.98 |
| DNME-41 | 99.00% | 96.07% | 96.18% | 3.82% | 96.12% | 97.57% | 1.00 | 1.00 | 1.00 | 0.99 | 27817.91 | 37.12 |
| DNME-42 | 100.00% | 96.16% | 96.30% | 3.70% | 96.23% | 98.12% | 1.00 | 1.00 | 1.00 | 1.00 | 29165.36 | 39.74 |
| DNME-43 | 100.00% | 95.83% | 96.00% | 4.00% | 95.91% | 97.96% | 1.00 | 1.00 | 1.00 | 1.00 | 32431.02 | 40.98 |
| DNME-44 | 100.00% | 95.93% | 96.09% | 3.91% | 96.00% | 98.00% | 1.00 | 1.00 | 1.00 | 1.00 | 29634.83 | 39.79 |
| DNME-45 | 100.00% | 96.64% | 96.75% | 3.25% | 96.70% | 98.35% | 1.00 | 1.00 | 1.00 | 1.00 | 29266.28 | 39.64 |
| DNME-46 | 75.51% | 96.48% | 95.55% | 4.45% | 96.11% | 84.36% | 0.98 | 0.98 | 0.98 | 0.74 | 40728.80 | 54.77 |
| DNME-47 | 53.06% | 97.24% | 95.05% | 4.95% | 96.46% | 68.10% | 0.98 | 0.98 | 0.98 | 0.52 | 37704.89 | 49.80 |
| DNME-48 | 100.00% | 96.18% | 96.32% | 3.68% | 96.24% | 98.12% | 1.00 | 1.00 | 1.00 | 1.00 | 29347.66 | 36.58 |
| DNME-49 | 100.00% | 96.66% | 96.77% | 3.23% | 96.72% | 98.36% | 1.00 | 1.00 | 1.00 | 1.00 | 29462.48 | 37.94 |
| DNME-50 | 92.00% | 96.67% | 96.50% | 3.50% | 96.58% | 94.20% | 1.00 | 1.00 | 1.00 | 0.92 | 27235.78 | 33.39 |
| DNME-51 | 100.00% | 95.90% | 96.06% | 3.94% | 95.97% | 97.99% | 1.00 | 1.00 | 1.00 | 1.00 | 29564.89 | 43.45 |
| DNME-52 | 100.00% | 96.12% | 96.26% | 3.74% | 96.19% | 98.09% | 1.00 | 1.00 | 1.00 | 1.00 | 26368.31 | 32.59 |
| DNME-53 | 100.00% | 96.04% | 96.19% | 3.81% | 96.11% | 98.06% | 1.00 | 1.00 | 1.00 | 1.00 | 28778.68 | 38.36 |
| DNME-54 | 100.00% | 96.47% | 96.59% | 3.41% | 96.54% | 98.27% | 1.00 | 1.00 | 1.00 | 1.00 | 27605.32 | 35.68 |
| DNME-55 | 100.00% | 96.21% | 96.35% | 3.65% | 96.28% | 98.14% | 1.00 | 1.00 | 1.00 | 1.00 | 27298.25 | 32.75 |
| DNME-56 | 100.00% | 95.76% | 95.93% | 4.07% | 95.84% | 97.92% | 1.00 | 1.00 | 1.00 | 1.00 | 30157.19 | 36.20 |
| DNME-57 | 100.00% | 96.17% | 96.31% | 3.69% | 96.24% | 98.12% | 1.00 | 1.00 | 1.00 | 1.00 | 30499.87 | 36.64 |
| DNME-58 | 100.00% | 95.42% | 95.62% | 4.38% | 95.50% | 97.76% | 1.00 | 1.00 | 1.00 | 1.00 | 26312.31 | 34.01 |
| DNME-59 | 100.00% | 95.72% | 95.90% | 4.10% | 95.80% | 97.91% | 1.00 | 1.00 | 1.00 | 1.00 | 29669.62 | 38.29 |
| DNME-60 | 100.00% | 95.98% | 96.13% | 3.87% | 96.05% | 98.03% | 1.00 | 1.00 | 1.00 | 1.00 | 29386.87 | 39.11 |
| DNME-61 | 100.00% | 95.70% | 95.88% | 4.12% | 95.78% | 97.90% | 1.00 | 1.00 | 1.00 | 1.00 | 27833.80 | 37.39 |
| DNME-62 | 100.00% | 96.23% | 96.37% | 3.63% | 96.30% | 98.15% | 1.00 | 1.00 | 1.00 | 1.00 | 30564.59 | 39.94 |
| DNME-63 | 100.00% | 95.83% | 96.00% | 4.00% | 95.91% | 97.96% | 1.00 | 1.00 | 1.00 | 1.00 | 29317.74 | 36.23 |
| DNME-64 | 100.00% | 97.04% | 97.13% | 2.87% | 97.10% | 98.54% | 1.00 | 1.00 | 1.00 | 1.00 | 28982.83 | 35.69 |
| DNME-65 | 100.00% | 96.68% | 96.79% | 3.21% | 96.74% | 98.37% | 1.00 | 1.00 | 1.00 | 1.00 | 30428.33 | 39.71 |
| DNME-66 | 100.00% | 96.66% | 96.77% | 3.23% | 96.72% | 98.36% | 1.00 | 1.00 | 1.00 | 1.00 | 28763.86 | 38.85 |
| DNME-67 | 100.00% | 96.23% | 96.37% | 3.63% | 96.30% | 98.15% | 1.00 | 1.00 | 1.00 | 1.00 | 30004.26 | 40.34 |
| DNME-68 | 100.00% | 95.42% | 95.62% | 4.38% | 95.51% | 97.76% | 1.00 | 1.00 | 1.00 | 1.00 | 26785.03 | 34.79 |
| DNME-69 | 100.00% | 96.01% | 96.16% | 3.84% | 96.08% | 98.04% | 1.00 | 1.00 | 1.00 | 1.00 | 30930.07 | 38.10 |
| DNME-70 | 100.00% | 95.65% | 95.83% | 4.17% | 95.73% | 97.87% | 1.00 | 1.00 | 1.00 | 1.00 | 29276.00 | 39.87 |
| DME-1 | 35.14% | 98.30% | 95.38% | 4.62% | 98.23% | 51.35% | 0.37 | 0.25 | 0.37 | 0.13 | 67088.08 | 137.29 |
| DME-2 | 0.00% | 98.51% | 0.00% | 100.00% | 98.39% | 0.00% | 0.42 | 0.39 | 0.43 | 0.00 | 66366.94 | 135.81 |
| DME-3 | 31.82% | 98.21% | 94.67% | 5.33% | 98.08% | 47.63% | 0.56 | 0.66 | 0.66 | 0.21 | 43360.83 | 93.74 |
| DME-4 | 4.88% | 98.27% | 73.85% | 26.15% | 98.04% | 9.15% | 0.56 | 0.82 | 0.82 | 0.04 | 23487.61 | 50.10 |
| DME-5 | 78.57% | 99.72% | 99.64% | 0.36% | 99.66% | 87.86% | 0.98 | 0.98 | 0.98 | 0.77 | 24765.78 | 37.21 |
| DME-6 | 98.99% | 97.85% | 97.87% | 2.13% | 97.85% | 98.43% | 0.99 | 0.99 | 0.99 | 0.98 | 9811.64 | 15.25 |
| DME-7 | 100.00% | 91.98% | 92.57% | 7.43% | 92.01% | 96.14% | 1.00 | 1.00 | 1.00 | 1.00 | 2122.37 | 3.06 |
| DME-8 | 100.00% | 70.67% | 77.32% | 22.68% | 70.78% | 87.21% | 1.00 | 1.00 | 1.00 | 1.00 | 2171.61 | 3.10 |
| DME-9 | 100.00% | 82.44% | 85.06% | 14.94% | 82.50% | 91.93% | 1.00 | 1.00 | 1.00 | 1.00 | 2366.83 | 3.67 |
| DME-10 | 100.00% | 87.09% | 88.56% | 11.44% | 87.13% | 93.93% | 1.00 | 1.00 | 1.00 | 1.00 | 2559.47 | 3.73 |
| DME-11 | 100.00% | 98.70% | 98.72% | 1.28% | 98.70% | 99.35% | 0.93 | 0.93 | 0.93 | 0.93 | 57318.15 | 89.89 |
| DME-12 | 100.00% | 96.93% | 97.02% | 2.98% | 96.94% | 98.49% | 1.00 | 1.00 | 1.00 | 1.00 | 3602.08 | 5.15 |

1. Zhang,Y. and Liu,J.S. (2007) Bayesian inference of epistatic interactions in case-control studies (Supplementary) Nat. Genet.,39, 1167–1173.
2. Peng-Jie Jing, Hong-Bin Shen (2014), [MACOED: A multi-objective ant colony optimization algorithm for SNP epistasis detection in genome-wide association studies (supplementary),](http://bioinformatics.oxfordjournals.org/content/31/5/634.short?rss=1) Bioinformatics, 2014.
3. Urbanowicz et al.: GAMETES: a fast, direct algorithm for generating pure, strict, epistatic models with random architectures. BioDataMining 2012 5:16.
4. Velez, D. R., White, B. C., Motsinger, A. A., Bush, W. S., Ritchie, M. D., Williams, S. M. and Moore, J. H. (2007[), A balanced accuracy function for epistasis modeling in imbalanced datasets using multifactor dimensionality reduction. Genet. Epidemiol](http://onlinelibrary.wiley.com/doi/10.1002/gepi.20211/suppinfo)(supplymentary)., 31: 306–315. doi: 10.1002/gepi.20211
5. [Velez DR](http://www.ncbi.nlm.nih.gov/pubmed/?term=Velez DR%5BAuthor%5D&cauthor=true&cauthor_uid=17323372)^1^, [White BC](http://www.ncbi.nlm.nih.gov/pubmed/?term=White BC%5BAuthor%5D&cauthor=true&cauthor_uid=17323372), [Motsinger AA](http://www.ncbi.nlm.nih.gov/pubmed/?term=Motsinger AA%5BAuthor%5D&cauthor=true&cauthor_uid=17323372), [Bush WS](http://www.ncbi.nlm.nih.gov/pubmed/?term=Bush WS%5BAuthor%5D&cauthor=true&cauthor_uid=17323372), [Ritchie MD](http://www.ncbi.nlm.nih.gov/pubmed/?term=Ritchie MD%5BAuthor%5D&cauthor=true&cauthor_uid=17323372), [Williams SM](http://www.ncbi.nlm.nih.gov/pubmed/?term=Williams SM%5BAuthor%5D&cauthor=true&cauthor_uid=17323372), [Moore JH](http://www.ncbi.nlm.nih.gov/pubmed/?term=Moore JH%5BAuthor%5D&cauthor=true&cauthor_uid=17323372). (2007), A balanced accuracy function for epistasis modeling in imbalanced datasets using multifactor dimensionality reduction. [Genet Epidemiol.](http://www.ncbi.nlm.nih.gov/pubmed/17323372) 2007 May;31(4):306-15.
6. <http://discovery.dartmouth.edu/epistatic_data/>
